# Photoresponsive Reversible Assembly of Helical Poly(isocyanide)s

**DOI:** 10.1021/acspolymersau.6c00119

**Published:** 2026-07-31

**Authors:** Jingyang Wang, Federica Kauffmann, Chengyuan Wang, Matthew D. Hannigan, Marcus Weck

**Affiliations:** Molecular Design Institute and Department of Chemistry, 5894 New York University, New York, New York 10003, United States

**Keywords:** helical polymers, poly(isocyanide)s, photoresponsive assembly, quadruple hydrogen bonding, azobenzene, postpolymerization modification, photoswitching

## Abstract

We report the synthesis of helical poly­(isocyanide)­s (PICs) functionalized with azobenzene-ureidopyrimidinone (Azo-Upy) motifs, designed to provide photoswitchable control over quadruple hydrogen-bonding interactions. The PICs were accessed via nickel-catalyzed polymerization with controlled molecular weights and low dispersities. With the photoisomerization of the *E*-Azo-Upy motif to form the (*Z*-Azo-Upy)_2_ dimer, the resulting polymers undergo reversible hydrogen bonding triggered by UV/blue light irradiation. The assembly can be tuned between intrachain collapse and interchain cross-linking by varying concentration or solvent. Circular dichroism (CD) spectroscopy confirms that the helical integrity of the PIC backbone is retained during the photoswitching process. Furthermore, an azide-functionalized Azo-Upy motif exhibits efficient photoresponsiveness to facilitate postpolymerization modification using click chemistry. The presented system establishes a robust platform for realizing controllable assembly of helical polymers to achieve higher-order structural hierarchies through photoresponsive hydrogen bonding.

## Graphical abstract

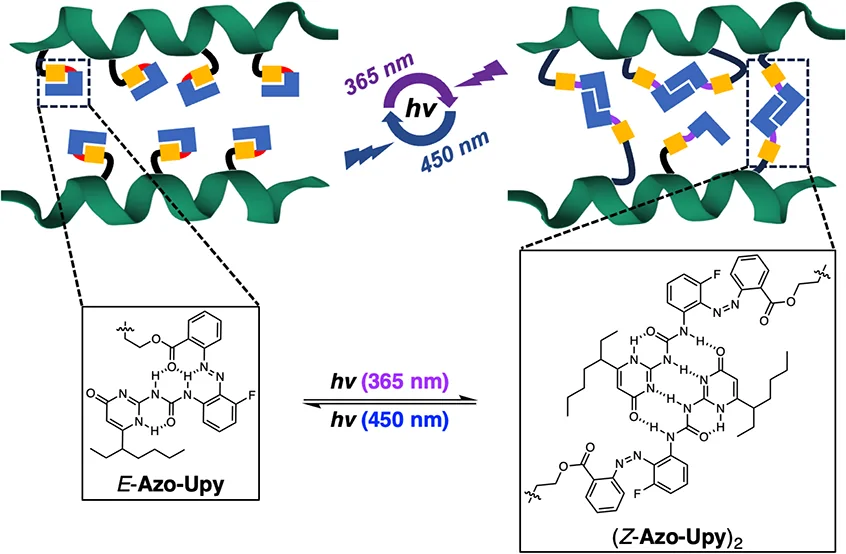

## Introduction

Nature exploits hierarchical self-assembly to construct functional materials with unparalleled complexity.^1^ For proteins, the precise organization of primary amino-acid sequences into secondary domains (e.g., α-helices and β-sheets) serves as a scaffold for the formation of higher-order architectures driven by orthogonal and/or synergistic noncovalent interactions such as hydrogen bonding, Coulombic interactions, and hydrophobic interactions, as well as dynamic covalent bonds (e.g., disulfide linkages).^2–4^ Upon folding, proteins exhibit a variety of functions—for example, catalysis, transport, and signaling—that have inspired chemists to develop synthetic analogues to emulate their complex yet delicate nature.^5,6^ In contrast to the limited monomer pool of natural peptides, the 22 natural α-amino acids, synthetic polymers possess the advantages of a wider monomer scope, enhanced tolerance to reaction conditions, a variety of polymerization methods, and facile scalability.^7^ Therefore, synthetic analogues to nature aim not only to enable the creation of advanced structures that do not exist in nature but also to broaden the scope of functions beyond the constraints of aqueous environments and specific pH conditions.^5,8–11^

To emulate the structural hierarchy and functional versatility of proteins, three strategies have been reported: single-chain polymeric nanoparticles,^12–16^ foldamers,^17,18^ and main-chain block copolymers.^8,19^ The main-chain block copolymer approach—a strategy developed by the Weck group—mimics protein by focusing on the modular incorporation of polymeric segments, where each block represents a distinct secondary structural domain.^8^ By employing controlled or living polymerization methods to ensure predetermined molecular weights and low dispersities, these building blocks can be systematically ligated, either covalently or through complementary molecular recognition units (MRUs) at the termini of polymer chains.^20–22^ Consequently, diverse main-chain block copolymers featuring higher-order architectures can be obtained with control in a “*plug and play*” fashion.^8,23–25^

Serving as the most abundant secondary domain in nature, the α-helix plays an indispensable role in sustaining the structural integrity and function of proteins.^26^ The development of synthetic analogues emulating helical polypeptides has garnered significant attention.^27^ Among the diverse helical synthetic analogues,^28,29^ poly­(isocyanide)­s (PICs) stand out as the first class of synthetic polymers reported to possess a stable helical conformation.^30^ Restricted by the steric hindrance of bulky side-chains and the lone-pair repulsion of imine nitrogen atoms along the backbone, PICs adopt a stable, rigid 4_1_ helical conformation with a high helix-inversion barrier.^31^ PICs were first polymerized by Millich,^32^ after which Nolte and Drenth et al. demonstrated that poly­(*tert-*butyl isocyanide)­s can be successfully resolved into discrete right- and left-handed helices without racemization, even at elevated temperatures.^33^ Currently, PICs are mostly prepared in a controlled manner via transition-metal catalyzed polymerization of isocyanides, during which a preferred handedness can be achieved either through the design of monomers with chiral subunits or through the presence of chiral catalysts or additives.^30,34–40^

A distinctive structural feature of PICs is their exceptionally high side-chain density, as there is one side-chain connected to every backbone atom. This renders PICs an optimal system for functionalization with MRUs along the helical backbone, establishing a foundation for engineering complex, protein-like architectures via hierarchical self-assembly.^30,31^ To demonstrate the utility of helical scaffolds, our group has reported the supramolecular assembly of side-chain functionalized PICs mediated by metal–ligand coordination and electrostatic phenyl–pentafluorophenyl quadrupole interactions.^41,42^ Building upon these foundations, an orthogonal supramolecular assembly system was subsequently developed integrating both metal coordination and hydrogen bonding.^26^ These advancements demonstrate the feasibility of emulating proteins’ tertiary structures through the assembly of side-chain functionalized PICs.^43^

While various external stimuli, ranging from pH^44,45^ and temperature^46,47^ to redox potential^48,49^ and competitive guests,^26,50^ have been used to modulate the assembly of synthetic polymers, light stands out as an ideal modality due to its nondestructive nature and unique spatiotemporal resolution.^12,51–54^ The implementation of photoresponsiveness to construct reversible assembly systems for helical polymers into higher-order architectures, however, remains underexplored.^29,30,55^ The 2-ureido-4­[1*H*]-pyrimidinone (Upy) motif, originally developed by the Meijer group, dimerizes via self-complementary quadruple hydrogen bonds (*K*_dim_ > 10^6^ M^–1^ in CHCl_3_).^56–58^ Recently, Wu and coworkers successfully realized the chain-end functionalization of star-shaped helical PICs with Upy motifs. By taking advantage of terminal quadruple hydrogen bonding, well-defined supramolecular polymer frameworks with higher-ordered structures have been constructed with tunable sizes and biomimicking applications.^59,60^ To introduce dynamic control to this hydrogen bonding interaction, Zhang and coworkers modified the Upy molecule with an *ortho*-ester substituted azobenzene (Azo) unit as the “photoswitch”.^61,62^ In response to UV or blue light irradiation, with the photoisomerization of the azobenzene structure, the obtained azobenzene-ureidopyrimidinone (Azo-Upy) motif is capable of unlocking/locking the hydrogen-bonding sites within the Upy unit, leading to the reversible formation/disassembly of intermolecular quadruple hydrogen-bonded dimers (*K*_dim_ = 1.2 × 10^5^ M^–1^ in CDCl_3_). We hypothesized that its side-chain integration onto the PIC backbone would facilitate the construction of helical polymers capable of reversible, photoresponsive assembly mediated by dynamic quadruple hydrogen bonds. Herein, by utilizing the Azo-Upy photoswitch, we address the challenge of decoupling dynamic hydrogen bonding interactions from static polymer architectures. We report the design and synthesis of helical poly­(isocyanide)­s functionalized with photoresponsive Azo-Upy side-chains and demonstrate that PIC copolymers can be accessed via nickel-catalyzed copolymerization with controlled molecular weights and low dispersities. *L*-menthol modified isocyanide (***L*** **-Men-PhNC**) is incorporated as a comonomer to enhance solubility and impart the preferred handedness of the helical scaffold. The resulting PIC copolymers exhibit reversible quadruple hydrogen bonding in response to UV or blue light irradiation. This dynamic process is characterized by nuclear magnetic resonance spectroscopy (NMR), ultraviolet–visible spectroscopy (UV–vis), circular dichroism (CD), and dynamic light scattering (DLS). We demonstrate that the preference between intrachain collapse and interchain cross-linking can be modulated in a concentration- and solvent-dependent manner. Furthermore, to broaden the applicability of our strategy, we introduce an azide-functionalized Azo-Upy motif (**Azo-Upy-N**_**3**_) to allow for postpolymerization modification under “click” conditions.

## Experimental Section

### General Materials

Unless otherwise noted, all reagents were purchased from commercial suppliers (Sigma-Aldrich, Oakwood Chemical, Fisher Scientific, TCI Chemical, and Alfa Aesar) and used without further purification. Flash column chromatography was performed using silica gel (Sorbtech, 200–400 mesh; Supelco, 70–230 mesh) or neutral aluminum oxide (Sigma-Aldrich) as the stationary phase.

### Instrumentation Information

Nuclear Magnetic Resonance (NMR): ^1^H NMR, ^13^C NMR, and ^19^F-NMR spectra were recorded at 25 °C on a Bruker AV4 500 MHz, or a Bruker AVIII 600 MHz. ^13^C NMR and ^19^F-NMR spectra were recorded with complete proton decoupling. All chemical shifts are reported in parts per million (ppm) downfield from tetramethylsilane (TMS) with the solvent resonance as the internal standard. Coupling constant (*J*) are reported in Hz and refer to apparent peak multiplicities. Gel-Permeation Chromatography (GPC): GPC characterizations were obtained using a Shimadzu pump coupled to Shimadzu UV and RI detectors with tetrahydrofuran (THF) as the mobile phase. The injection volume was 75 μL and the flow rate was 1.0 mL/min on a Shodex GPC column set (KF-G 4A as the guard column, KF-804, and KF-802.5) at 40 °C. The GPC instrument was calibrated using poly­(styrene) standards (EasiCal PS-2, Agilent Technologies, Santa Clara, CA). *M*_w_, *M*_n_, and *Đ* represent weight-average molecular weight, number-average molecular weight, and dispersity index, respectively. Liquid Chromatography Time-Of-Flight Mass Spectrometry (LC-TOF-MS): High-resolution mass spectra of samples in methanol were acquired with an Agilent 6224 TOF LC/MS mass spectrometer using an electrospray ionization (ESI) ion-source. The samples were separated and analyzed by an Agilent 1260 Infinity HPLC equipped with CHIRALPAK IA-3 column. Ultraviolet–Visible Spectroscopy (UV–vis): UV–vis spectra were recorded on an Agilent Cary 3500 UV–vis spectrometer using absorbance mode in a quartz cuvette with 1.0 cm path length collecting within the wavelength range of 800 to 200 nm at 25 °C. Fourier Transform Infrared Spectroscopy (FT-IR): FT-IR spectra were recorded on a Thermo Fisher Scientific Nicolet iS50 FT-IR spectrometer, equipped with a Smart iTX with monolithic diamond plate ATR. Circular Dichroism Spectroscopy (CD): CD spectra were taken on a Jasco J-1500 Circular Dichroism spectrometer in a quartz cuvette with 0.1 cm path length at 25 °C. CD spectra were corrected against a blank solvent background to remove aberrations from the solvent and cuvette—spectra containing samples were taken in the same cuvette as the background spectrum was collected in. Dynamic Light Scattering (DLS): Hydrodynamic diameters of helical poly­(isocyanide)­s before and after UV/blue light irradiation were determined at 25 °C by DLS using Malvern Zetasizer Nano series with a 633 nm laser module.

### General Procedure for Polymerization

In a typical polymerization, monomers **M1** (40.0 mg, 49.5 μmol) and **M2** (28.3 mg, 99.0 μmol) were dissolved in dry dichloromethane (DCM) (2.50 mL) in a Schlenk flask. Initiator **I2** (3.28 mg, 4.95 μmol) was dissolved in dry DCM (0.470 mL) and added to the stirring solution of the monomers under a N_2_ atmosphere. After stirring at room temperature for 1 h, the solvent was removed under reduced pressure. The crude product was redissolved in a minimal amount of chloroform (CHCl_3_) and precipitated into a cold mixture of MeOH/H_2_O (3:1 v/v, 20.0 mL). The polymer product was collected by filtration, washed with additional cold MeOH/H_2_O (3:1 v/v, 20.0 mL), and dried under vacuum as a brown solid **P1a** (53.0 mg, 74%).

### General Procedure for Postpolymerization Modification (PPM)

In a typical PPM procedure, the precursor polymer **P3** was prepared under the polymerization conditions described above with **M3** as the sole monomer. Then, compound **5** (129 mg, 183 μmol) and PMDETA (79.4 mg, 458 μmol) were added to a stirring solution of **P3** (20.0 mg, 91.6 μmol as of alkyne units) in dry DCM in a Schlenk flask. Three freeze–pump–thaw cycles were performed to degas the system, after which CuBr (26.4 mg, 183 μmol) was added in one portion under an argon flow. The reaction mixture was stirred at room temperature for 72 h followed by filtration to remove insoluble copper salts. The solvent was removed under reduced pressure. The crude residue was redissolved in a minimal amount of CHCl_3_ and precipitated into a cold mixture of MeOH/H_2_O (3:1 v/v, 20.0 mL). The resulting solid was collected by filtration, washed with additional cold MeOH/H_2_O (3:1 v/v, 10.0 mL), and dried under vacuum to afford **P3P** as an orange solid (34.7 mg, 41%).

### General Procedure for Irradiation Processes

In a typical irradiation procedure, solutions of photoresponsive small molecules or polymers in CHCl_3_, tetrahydrofuran (THF), or CDCl_3_ were prepared at defined concentrations. The solutions were subjected to irradiation for 5 min while maintaining a temperature of 25 °C. A 365 nm LED lamp from EvoluChem (SKU: HCK1012-01-029, 18 W, 190 mW/cm^2^, 50/60 Hz) and a 450 nm LED lamp from EvoluChem (SKU: HCK1012-01-002, 18 W, 328 mW/cm^2^, 50/60 Hz) were used to perform UV and blue light irradiation, respectively.

## Results and Discussion

### Synthesis of Monomers and Polymers

We synthesized the Azo-Upy modified isocyanide monomer, **M1** (**Azo-Upy-PhNC**), as shown in Scheme 1A. A dodecyl spacer was introduced to enhance solubility and to mitigate the steric hindrance imposed by the bulky Azo-Upy motif on the helical PIC backbone. The alcohol precursor **1** and formamide **2** were synthesized following literature procedures.^61,63^ Subsequent Steglich esterification between **1** and **2** afforded the formamide intermediate **3**. The modest overall yield of **M1** (20% from **1**) is primarily attributed to the challenging dehydration of intermediate **3**. Screening for optimal dehydration conditions proved nontrivial as the multiple Lewis basic sites within the Azo-Upy motif exhibited poor compatibility with conventional dehydrating reagents, leading to decomposition. The target monomer **M1** was ultimately obtained using diphosgene as the dehydrating agent and *N*-methylmorpholine as the base at low temperatures. The preparation of the nickel initiators commenced with commercially available and air-stable Ni­(ClO_4_)_2_·6H_2_O. Following a literature procedure,^64^ the square-planar initiator **I1** was synthesized via coordination with *tert*-butyl isocyanide. Initiator **I1** was subsequently modified with *n*-butylamine to yield **I2** to enhance its nucleophilicity (Scheme 1B). Successful modification is confirmed by the appearance of N–C–N carbene stretching signal in the IR spectrum (Figure S1). To induce a preferred helical handedness within the polymer backbone, a chiral *L*-menthol derived monomer **M2** (***L*** **-Men-PhNC**) was used as the comonomer for **M1**, as depicted in Scheme 1. The nickel-catalyzed copolymerization was conducted in DCM at room temperature to afford the target copolymer **P1** (***L*** **-Men-Azo-Upy-PIC**) (Scheme 1C). The GPC characterization data of the copolymers are summarized in Table 1.

**Scheme 1:**
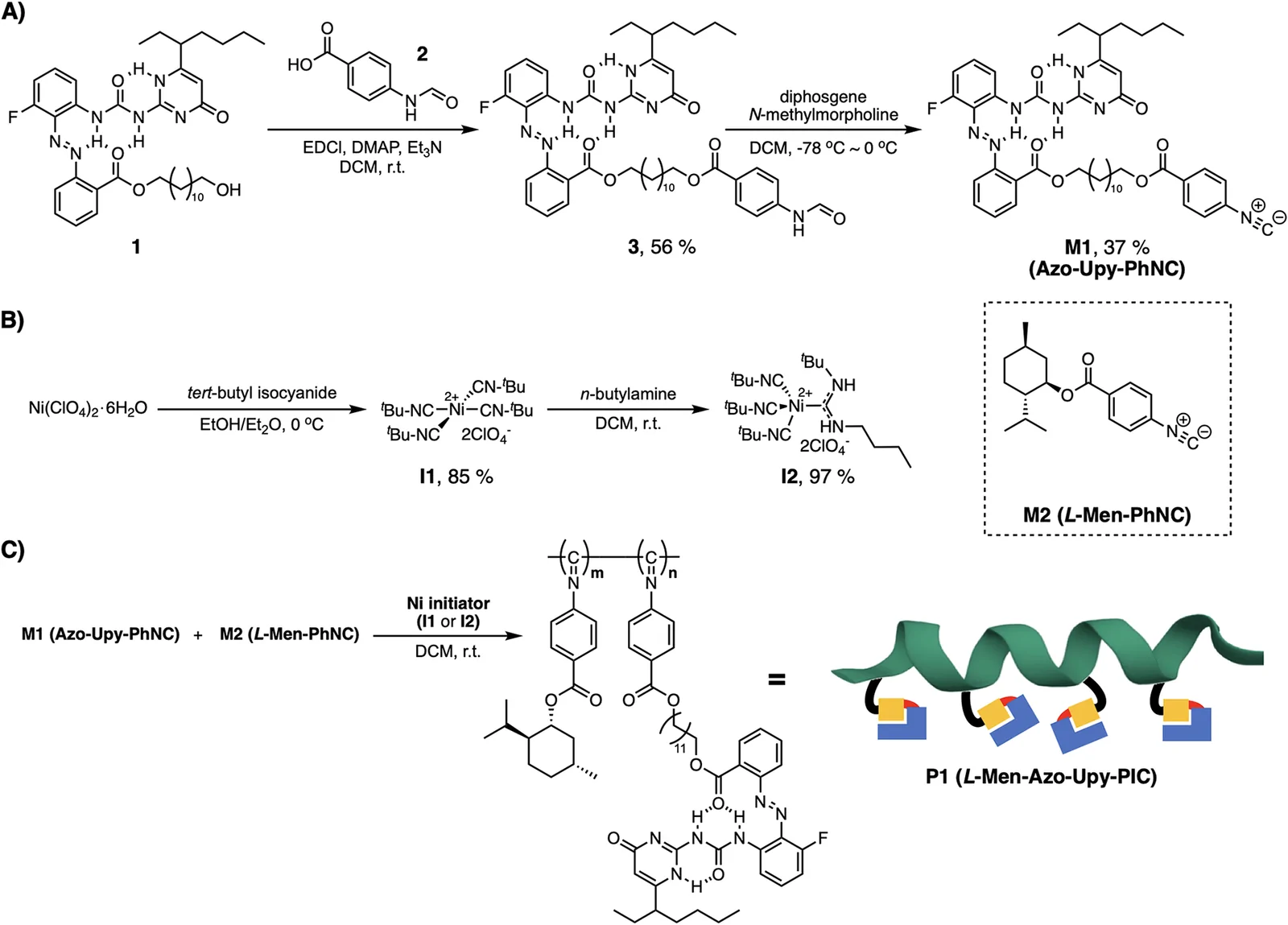
Synthetic Route of (A) Azo-Upy Functionalized Isocyanide Monomer **M1** (**Azo-Upy-PhNC**) (B) Nickel Initiators and (C) the Copolymer **P1** (***L*** **-Men-Azo-Upy-PIC**)

**Table 1:** GPC Data of Copolymers **P1a**-P1i^a^

Entry	Initiator	[M1]/[M2]/[I]	*M*_n_ (GPC) (kDa)	*Đ*	Theoretical *M*_n_ (kDa)
**P1a**	**I2**	10/20/1	13.0	1.12	14.4
**P1b**	**I2**	4/16/1	9.5	1.14	8.5
**P1c**	**I2**	4/36/1	11.2	1.15	14.2
**P1d**	**I2**	5/10/1	9.0	1.11	7.6
**P1e**	**I2**	15/30/1	15.7	1.18	21.3
**P1f**	**I2**	30/60/1	25.6	1.37	42.0
**P1g**	**I2**	40/80/1	32.6	1.56	55.8
**P1h**	**I2**	10/90/1	21.5	1.20	34.4
**P1i**	**I1**	10/90/1	24.3	1.26	34.4

a (Eluent THF; poly­(styrene) standards).

We used **I2** to synthesize a series of copolymers with varying [**M1**]/[**M2**] ratios. All copolymers (**P1a**–**P1c**) exhibit well-defined molecular weights and low dispersities (*Đ*) (Figure 1A). Although a higher content of the photoactive **M1** is desirable for the efficiency of the photoresponsive hydrogen bonding, we did not increase its ratio further to ensure solubility of the resulting helices. The copolymer with an [**M1**]/[**M2**] ratio of 1/2 was selected to investigate the livingness of the polymerization via the adjustment of the [M]/[I] ratio (**P1a**, **P1d**–**P1g**). The deviation of GPC-determined *M*_n_ from theory, which overestimated at low degrees of polymerization (DPs) and underestimated at high DPs, is attributed to the rigid, rod-like helical backbone of the PICs.^30^ Compared to the flexible random-coil configuration of poly­(styrene) standards, these rigid rods occupy larger hydrodynamic volumes for a given chain length but become comparatively more compact at higher-molecular-weights, as previously observed for PIC systems.^26,55^ For DPs up to 45 (**P1d**, **P1a**, **P1e**), the polymerization remains well-controlled, obtaining copolymers with low dispersities. As the target DP further increases, however, higher dispersities are observed (**P1f** and **P1g**). For copolymers **P1a**–**P1h**, the GPC-determined *M*_n_ exhibits a linear dependence on the theoretical *M*_n_, indicating control over molecular weights through variations of the [M]/[I] ratio (Figure 1B). At higher DPs, however, the increased dispersities indicate that the system deviates from livingness. Although this could be from a number of sources, it is likely due to the decreased solubility of the polymer at higher molecular weights, which leads to higher molecular weight species “crashing out” of solution or aggregating, preventing further extension. The narrow dispersities and a linear relationship between theoretical and observed *M*_n_ for polymers, however, indicate that we are able to achieve molecular weight control up to 40 kDa. Comparison of the initiators under identical conditions reveals that the amine-modified **I2** affords better control over dispersities compared to the unmodified **I1** (**P1h** and **P1i**). Polymer **P1i** displays a high-molecular-weight shoulder in its GPC traces (Figure 1A), which we suggest is due to aggregation. Attempts to eliminate this aggregation by using DMF as the GPC eluent were unsuccessful due to the insolubility of the copolymers in DMF.

**Figure 1:**
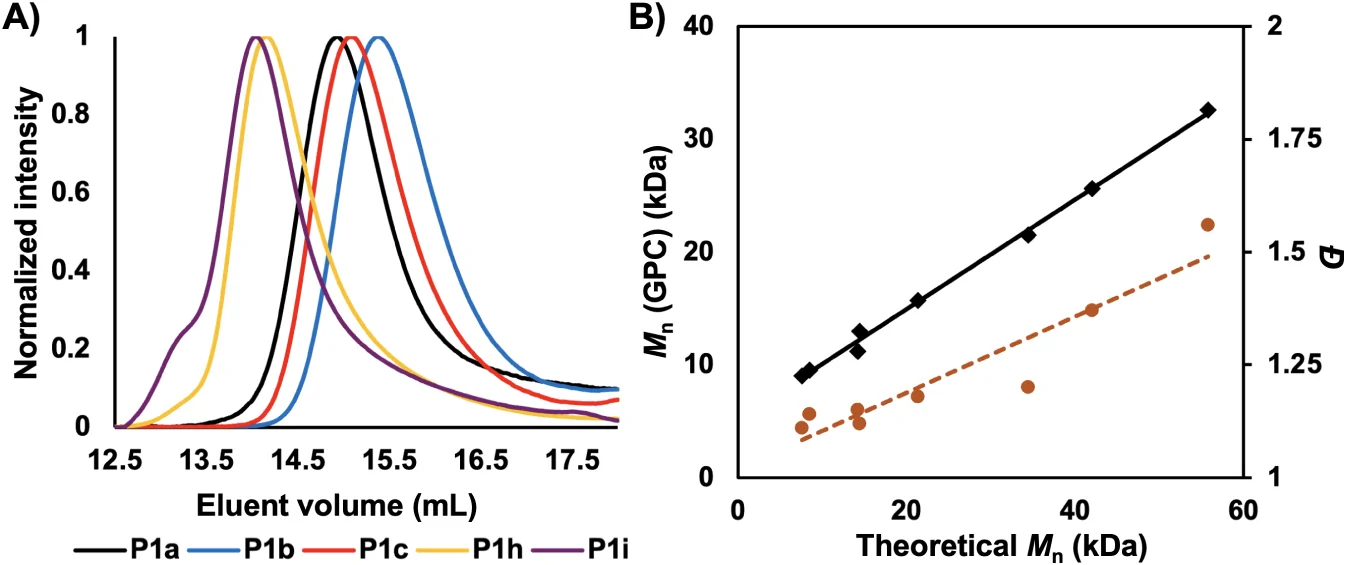
(A) GPC traces of copolymers **P1a** (black), **P1b** (blue), **P1c** (red), **P1h** (yellow), and **P1i** (purple). (B) Plot of the GPC-determined *M*_n_ (black) and *Đ* (orange) as a function of the theoretical *M*_n_ for copolymers **P1a**–**P1h**.

### Photoresponsive Assembly

With the functionalized copolymer **P1** in hand, we investigated its photoresponsive hydrogen bonding behavior. As reported by Zhang and coworkers, the Azo-Upy motif possesses a moderate dimerization constant (*K*_dim_ = 1.2 × 10^5^ M^–1^ in CDCl_3_),^61^ which is well-suited for establishing the dynamic equilibrium necessary for reversible assembly. Consequently, we anticipated that the formation and dissociation of intermolecular quadruple hydrogen bonds from the Azo-Upy unit—regulated by the *E*-to-*Z* or *Z-*to*-E* photoisomerization of the azobenzene structure upon UV or blue light irradiation—can lead to the photoresponsive assembly and disassembly of **P1** (Scheme 2). The assembly step is expected to proceed via a competition between interchain cross-linking and intrachain association.

**Scheme 2:**
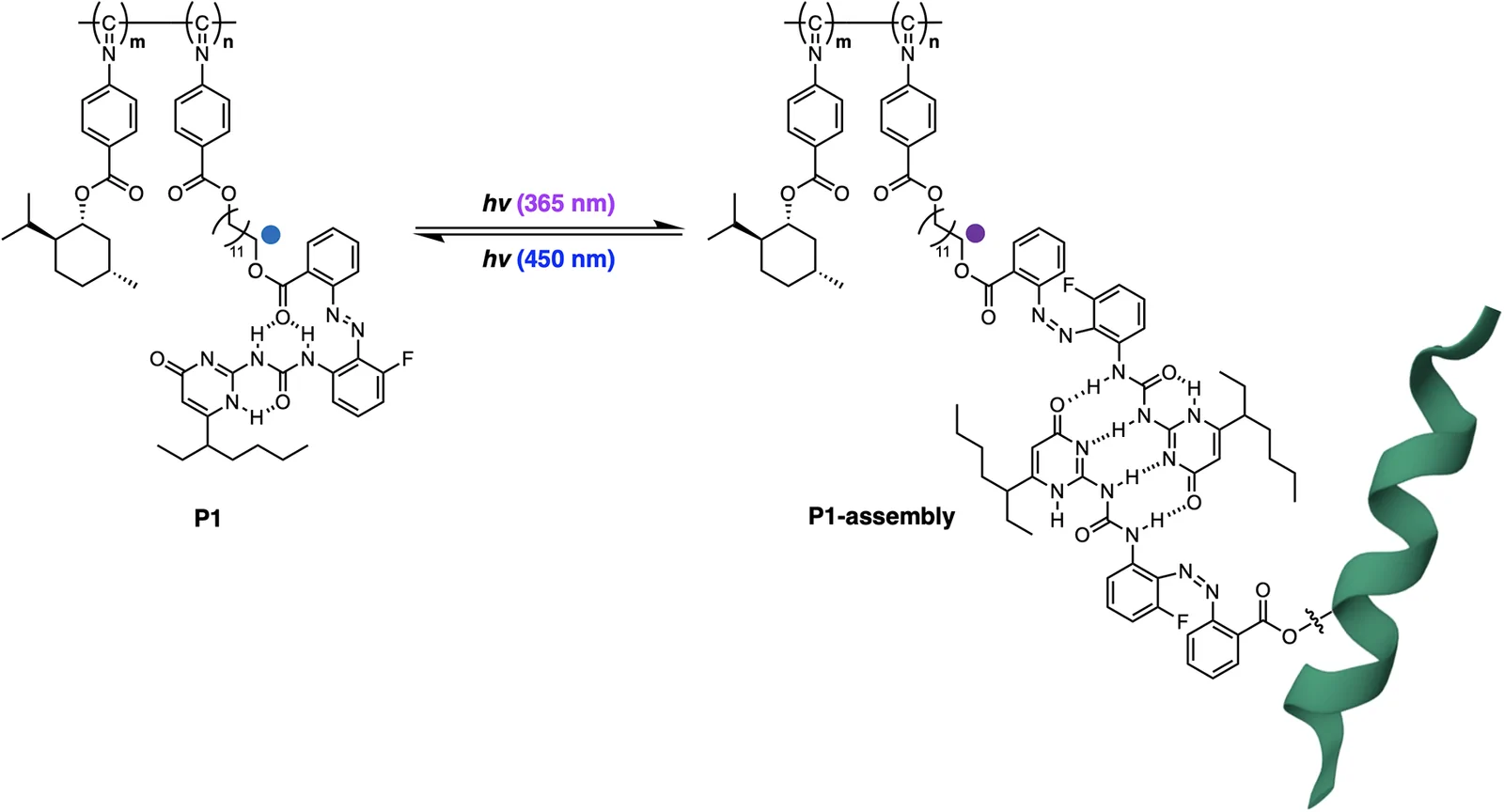
Photoresponsive Assembly of **P1** via Quadruple Hydrogen Bonds (Blue Dot: the Methylene Protons Adjacent to the Azo-Upy Unit Before Irradiation; Purple Dot: the Corresponding Protons After UV-Irradiated Assembly)

We first compared the GPC traces of **P1a** throughout the irradiation process (Figure 2A). The traces exhibit nearly identical peak shapes and similar retention times under standard GPC running conditions, confirming the structural integrity of the PIC backbone and that no detectable chain scission occurs upon irradiation. After UV irradiation (λ = 365 nm), the dispersity slightly increases from *Đ* = 1.12 ± 0.01 to *Đ* = 1.15 ± 0.02. Subsequent blue light irradiation (λ = 450 nm) of **P1a** partially restores the GPC trace (*Đ* = 1.13 ± 0.01). We further investigated the photoresponsiveness of **P1a** using ^1^H NMR spectroscopy (Figure 2B). Before irradiation, the spectrum of **P1a** displays broad signals, a characteristic feature of the rigid, closely packed helical backbone of PICs. Upon UV irradiation (λ = 365 nm), the system reaches a photostationary state (PSS), accompanied by the appearance of new signals from hydrogen bonding. Notably, the −CH_2_– protons adjacent to the Azo-Upy unit (as marked in Scheme 2) shift upfield by ∼0.3 ppm as a result of the *E*-to-*Z* photoisomerization of the azobenzene unit which disrupts the intramolecular hydrogen bonds in **P1** and leads to intermolecular quadruple hydrogen bonds in **P1-assembly**. Subsequent irradiation of **P1a** with blue light (λ = 450 nm) reverses the photoisomerization, confirming the reversibility of the assembly/disassembly process. To optimize the copolymer composition for maximum photoefficiency, we compared the assembly behavior of **P1a**, **P1b**, and **P1c** under identical UV irradiation conditions. In the ^1^H NMR spectra (Figure S2), a quantitative analysis of the integration ratios between the −CH_2_– signals corresponding to **P1** and **P1-assembly** confirms that **P1a** exhibits the highest isomerization ratio (1:0.80), significantly surpassing that of **P1b** (1:0.52) and **P1c** (1:0.35). The data suggest that a higher content of the photoactive Azo-Upy motif facilitates a more efficient photoswitching response. Therefore, polymer **P1a** was selected as the copolymer standard for all subsequent photoresponsiveness studies.

**Figure 2:**
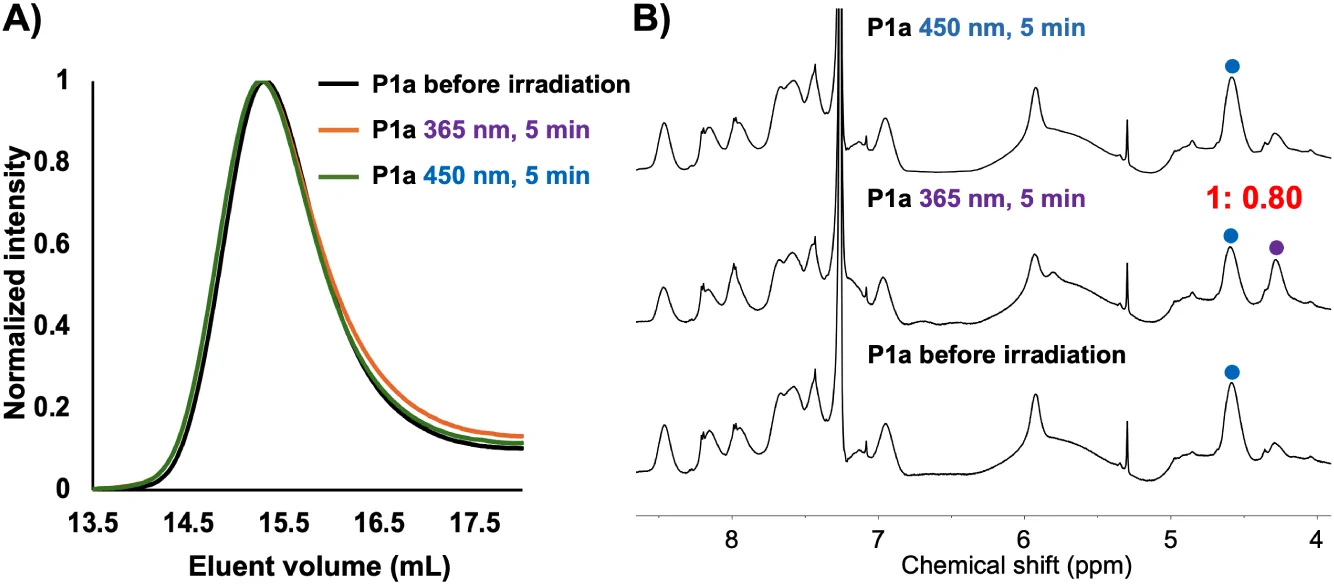
(A) GPC traces and (B) stacked partial ^1^H NMR spectra (600 MHz, 7.5 mg/mL in CDCl_3_ at 25 °C) of **P1a** before irradiation, after UV irradiation, and after subsequent blue light irradiation.

The UV–vis spectra of **P1a** provide further insights into the reversibility of the hydrogen bonding (Figure 3A). Before irradiation, **P1a** exhibits a peak absorbance (λ_max_) at 344 nm, characteristic of the π-π* transition of the *E*-azobenzene module within the Azo-Upy unit. Upon UV irradiation (λ = 365 nm), a significant decrease in absorbance at this wavelength is observed. This hypochromic effect is attributed to the formation of polymeric assembly, where the intermolecular cross-linking of the Azo-Upy motifs via quadruple hydrogen bonds reduces the molar absorptivity of the closely stacked azobenzene units.^65,66^ Upon subsequent blue light irradiation (λ = 450 nm), the absorbance intensity is partially recovered. This confirms the disassembly process and the reversibility of the system, consistent with the ^1^H NMR results in Figure 2B. Next, we investigated by CD spectroscopy whether the helical handedness is retained during the assembly process. Copolymer **P1a** exhibits a positive Cotton effect at approximately 360 nm before irradiation (Figure 3B), confirming the right handedness of the helical PIC.^31^ The CD spectra recorded after UV (λ = 365 nm) and subsequent blue light (λ = 450 nm) irradiations display negligible deviations from that before irradiation, indicating that the helical PIC backbone remains intact and conformationally stable throughout the irradiation process. This result further underscores that the photoresponsive assembly is driven exclusively by side-chain interactions without perturbing the rigid helical scaffold.

**Figure 3:**
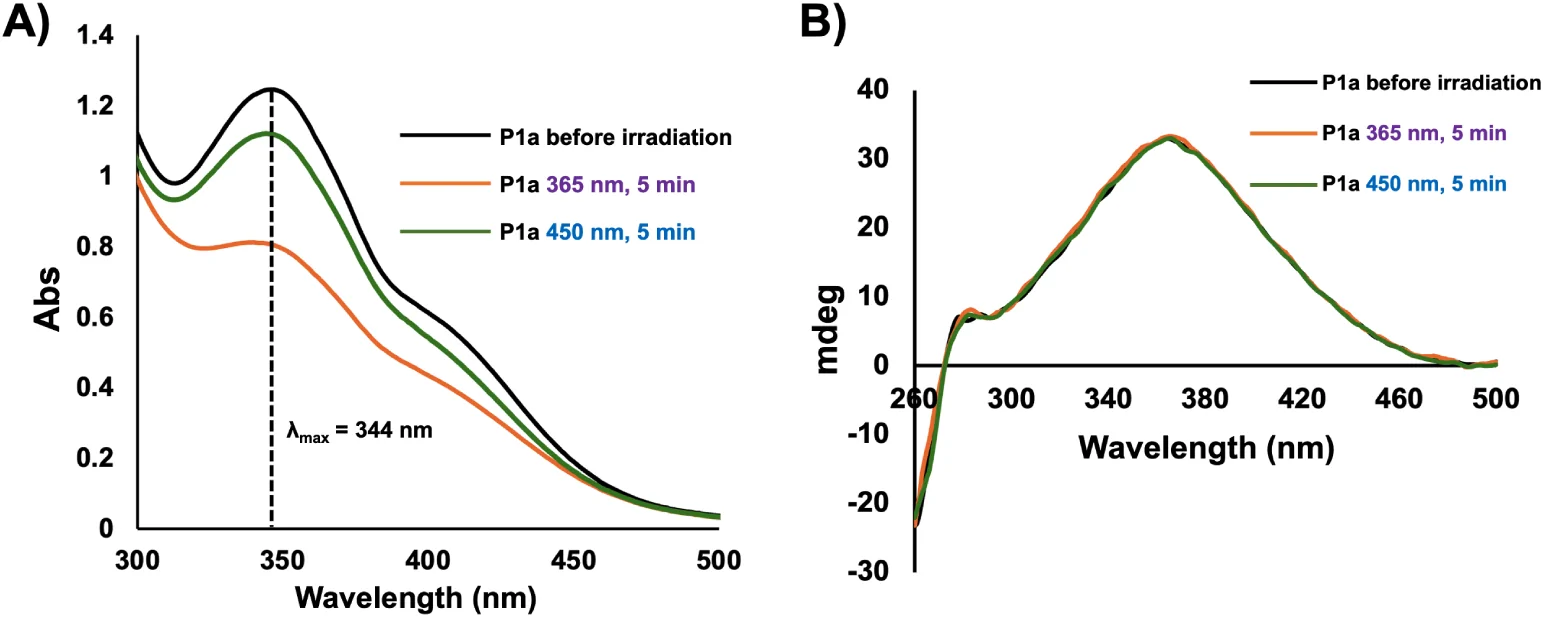
(A) UV–vis and (B) CD spectra of **P1a** before irradiation, after UV irradiation, and after subsequent blue light irradiation (UV–vis: 0.10 mg/mL of **P1a** in CHCl_3_ at 25 °C, l = 1.0 cm; CD: 0.50 mg/mL of **P1a** in CHCl_3_ at 25 °C, l = 0.1 cm).

Upon confirming the reversibility and backbone integrity of the photoresponsive supramolecular assembly, we proceeded to investigate the size variations of **P1a** throughout the irradiation process using DLS. The obtained hydrodynamic diameters (*D*_h_) are summarized in Table 2 (by intensity) and Table S1 (by volume). In CHCl_3_ before irradiation, the size distributions by intensity of **P1a** (Figure 4A and 4B) exhibit a primary peak around 7–8 nm and a broader peak (>100 nm), indicating the formation of partial aggregation. According to Rayleigh scattering theory,^67^ however, the scattering intensity of particles is proportional to the sixth power of their diameters (*I* ∝ *d* ^6^). Consequently, while the signal proportion of these larger aggregates is exaggerated in the intensity profiles, their actual volume fraction is negligible as confirmed in Figure S3A and S3B. The photoresponsive assembly of **P1a** in CHCl_3_ is found to be concentration-dependent. At a low concentration (1.0 mg/mL), UV irradiation (λ = 365 nm) causes the peak *D*_h_ to decrease from 7.2 to 5.6 nm (Table 2 and Figure 4A), indicating that quadruple hydrogen bonding drives intrachain collapse. Conversely, at a high concentration (10.0 mg/mL), UV irradiation (λ = 365 nm) leads to an increase in *D*_h_ from 8.0 to 10.7 nm (Figure 4B), demonstrating that interchain cross-linking overrides the intrachain collapse. Similar trends in the variations of *D*_h_ at different concentrations in CHCl_3_ were observed by Zhang and coworkers in a linear copolymer functionalized with Azo-Upy photoswitches.^61^ Subsequent blue light irradiation (λ = 450 nm) restores the *D*_h_ toward the values of the initial state, further proving the reversibility of the assembly. When THF is used as a hydrogen-bond acceptor solvent, the overall extent of initial aggregation is reduced (Figure 4C and 4D). At 1.0 mg/mL of **P1a** in THF, UV irradiation (λ = 365 nm) results in a marginal increase in *D*_h_ (from 7.7 to 8.6 nm, Table 2 and Figure 4C), which can be interpreted as a counterbalance where interchain cross-linking and intrachain collapse roughly offset each other. At a higher concentration (10.0 mg/mL), the *D*_h_ still increases from 8.0 to 9.4 nm upon UV irradiation (λ = 365 nm) (Figure 4D), confirming that interchain cross-linking remains dominant at elevated concentrations. The trends are consistent between the size distributions by intensity and by volume (Tables 2 and S1, Figures 4 and S3). These results provide further evidence for the photoresponsive reversible assembly process of **P1a**, where intrachain collapse and interchain cross-linking can be modulated by polymer concentration and/or the solvent.

**Table 2:** Hydrodynamic Diameters of **P1a** Measured by DLS (Size Distributions by Intensity)^a^

Sample	Solvent	Conc. (mg/mL)	*D*_h_ (nm)
**P1a** before irradiation	CHCl_3_	1.0	7.2 ± 1.5
**P1a** 365 nm, 5 min	CHCl_3_	1.0	5.6 ± 1.9
**P1a** 450 nm, 5 min	CHCl_3_	1.0	7.2 ± 1.5
**P1a** before irradiation	CHCl_3_	10.0	8.0 ± 2.4
**P1a** 365 nm, 5 min	CHCl_3_	10.0	10.7 ± 2.5
**P1a** 450 nm, 5 min	CHCl_3_	10.0	8.2 ± 2.5
**P1a** before irradiation	THF	1.0	7.7 ± 2.0
**P1a** 365 nm, 5 min	THF	1.0	8.6 ± 2.3
**P1a** 450 nm, 5 min	THF	1.0	8.1 ± 2.2
**P1a** before irradiation	THF	10.0	8.0 ± 2.1
**P1a** 365 nm, 5 min	THF	10.0	9.4 ± 2.5
**P1a** 450 nm, 5 min	THF	10.0	8.0 ± 2.3

a (*D*_h_ measured at 25 °C).

**Figure 4:**
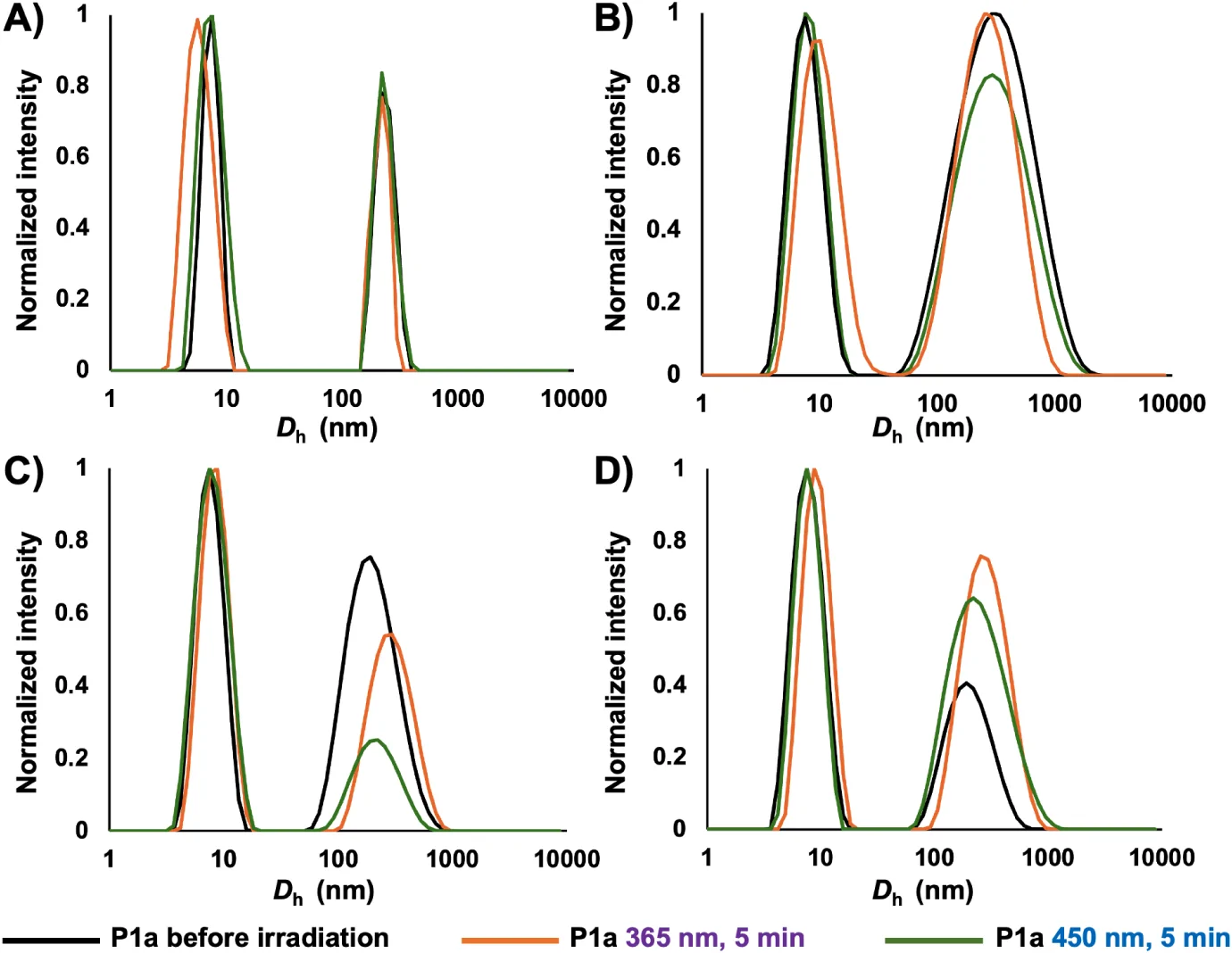
DLS profiles (size distributions by intensity) of **P1a** before irradiation, after UV irradiation, and after subsequent blue light irradiation at (A) 1.0 mg/mL in CHCl_3_, (B) 10.0 mg/mL in CHCl_3_, (C) 1.0 mg/mL in THF, and (D) 10.0 mg/mL in THF at 25 °C.

### A “Clickable” Azo-Upy Unit

While we successfully synthesized **P1** and confirmed its photoresponsive reversible assembly via quadruple hydrogen bonds, the preparation of **M1** presented synthetic challenges and resulted in low yields. Specifically, the final dehydration of formamide **3** to access the isocyanide group proves difficult, and the resulting monomer **M1** exhibits limited stability. To overcome these limitations and to render the Azo-Upy motif as a more universally usable photoswitch, we envisioned modifying it into a “clickable” unit. This strategy not only provides a more robust route for attaching the motif onto polymer chains via postpolymerization modification (PPM) but also opens up possibilities for potential chain-end functionalization. The feasibility of this methodology is supported by previous reports demonstrating successful PPM^68–70^ and chain-end functionalization^64^ on PICs using click chemistry. We designed the azide-functionalized Azo-Upy motif, **5** (**Azo-Upy-N**_**3**_), and synthesized it as outlined in Scheme 3. Starting from the literature-reported alcohol precursor **1**,^61^ efficient bromination under Appel conditions^71^ followed by the treatment with sodium azide at room temperature afforded the desired compound **5** with high yields under mild conditions.

**Scheme 3:**
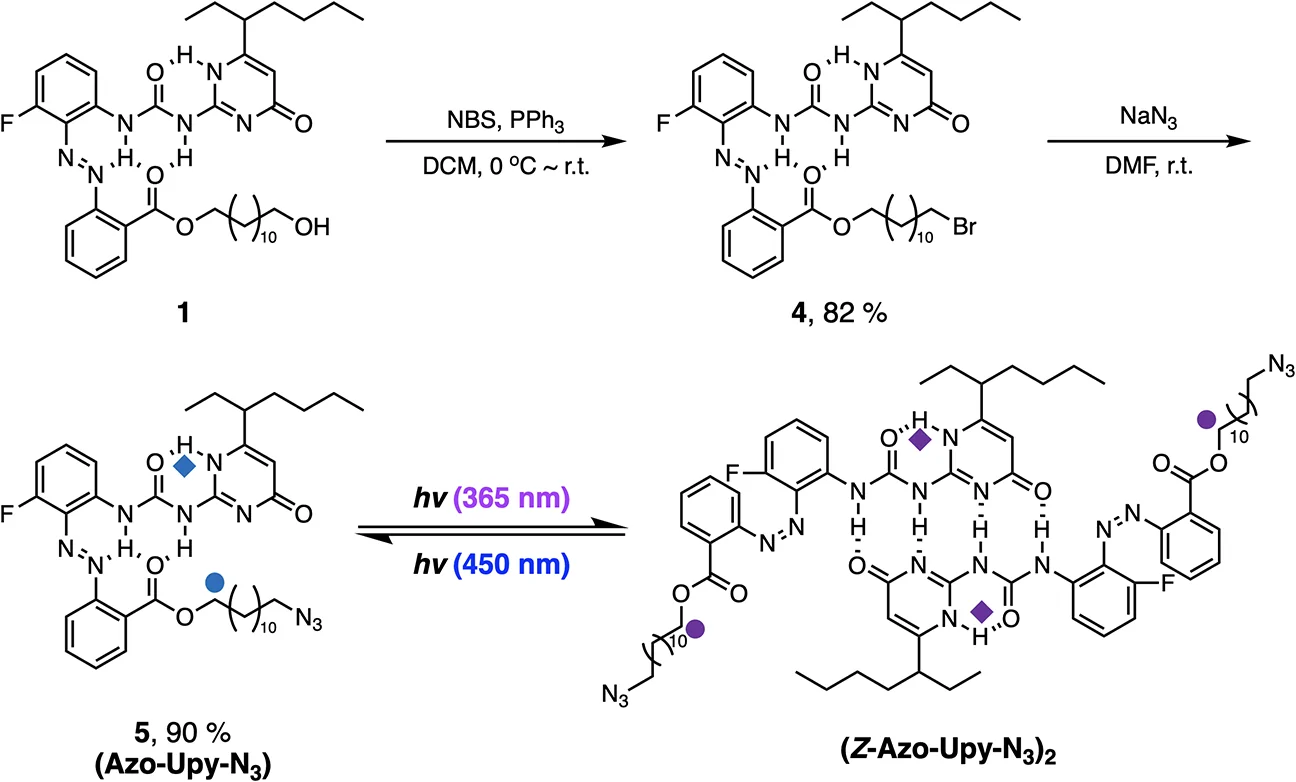
Synthesis of Compound **5** (**Azo-Upy-N**_**3**_) and Its Reversible Dimerization Behavior upon Irradiation (Blue Dot: the Featured Protons in **5** before Irradiation; Purple Dot: Corresponding Protons in (*Z*-**Azo-Upy-N**_**3**_)_2_ after Dimerization)

Following the successful synthesis of **5**, we investigated its photoresponsive self-dimerization behavior. UV–vis absorption spectra of **5** before and after irradiation with different wavelengths were recorded (Figure S4A) with a peak absorbance (λ_max_) at 346 nm, closely mirroring the optical properties of **P1a** (λ_max_ = 344 nm, in Figure 3A). The optimal irradiation wavelengths were determined as λ = 365 nm for dimerization and λ = 450 nm for reverse disassembly, respectively, since the highest isomeric ratios of **5** at PSS are achieved upon irradiation with these wavelengths. Clickable unit **5** is proven to exhibit excellent light fatigue resistance as evidenced through cycling tests under optimal irradiation conditions (Figure S4B).

To provide structural insight into its self-dimerization process, ^1^H NMR spectra of **5** were recorded before irradiation, after UV irradiation, and after subsequent blue light irradiation, respectively (Figure 5). Before irradiation, **5** adopts the thermodynamically stable *E*-**Azo-Upy-N**_**3**_ configuration. Upon UV irradiation (λ = 365 nm), with the dimerization of **5** to form (*Z*-**Azo-Upy-N**_**3**_)_2_, the N–H protons from the Upy unit undergo a downfield shift while the −CH_2_– protons adjacent to the Azo-Upy unit shift upfield (as marked in Scheme 3). Based on the integration of the shifted −CH_2_– signals, the isomeric ratio of **5** at PSS was calculated to be approximately *E*/*Z* = 1:0.52. Subsequent blue light irradiation (λ = 450 nm) effectively disrupts the dimerization, driving the system back to a PSS that closely resembles the unirradiated state. Time-dependent ^1^H NMR tracking revealed that a 5 min irradiation is optimal to reach the PSS under both wavelengths, as irradiation beyond 5 min yielded no further changes in the isomeric ratios (Figure S5). To confirm the formation of the (*Z*-**Azo-Upy-N**_**3**_)_2_ dimer and to quantify the size of **5** after UV irradiation, diffusion-ordered spectroscopy (DOSY) experiments were performed. Before irradiation, **5** (**Azo-Upy-N**_**3**_) is found to diffuse as one entity with a single diffusion coefficient *D* = 1.59 × 10^–9^ m^2^s^–1^ (Figure S6). Following UV irradiation (λ = 365 nm), two distinct diffusing entities are resolved from the dimerization of **5** with diffusion coefficients of *D*_1_ = 1.21 × 10^–9^ m^2^s^–1^ and *D*_2_ = 9.18 × 10^–10^ m^2^s^–1^ (Figure S7). We assign these to the *E*-**Azo-Upy-N**_**3**_ and the formed (*Z*-**Azo-Upy-N**_**3**_)_2_ dimer, respectively. Based on the ratio of *D*_2_/*D*_1_ = 75.9%, according to the Stokes–Einstein equation,^72^ the volume of the diffusing entity formed by the (*Z*-**Azo-Upy-N**_**3**_)_2_ dimer can be estimated as 2.3 times bigger than that of the *E*-**Azo-Upy-N**_**3**_, further supporting the self-dimerization behavior of **5** through intermolecular quadruple hydrogen bonds upon UV irradiation.^61^ Additionally, the formation of the quadruple hydrogen-bonded (*Z*-**Azo-Upy-N**_**3**_)_2_ dimer is highly sensitive to solvent polarity. When a small amount of DMSO-*d*_6_ was added to the UV-irradiated CDCl_3_ solution of **5**, both, the disappearance of the dimer signals and the appearance of the *Z*-**Azo-Upy-N**_**3**_ monomer signals were observed (Figure S8). This specific disruption by a competitive solvent is consistent with the previous report,^61^ which confirms the dynamic hydrogen-bonding nature of the assembly.

**Figure 5:**
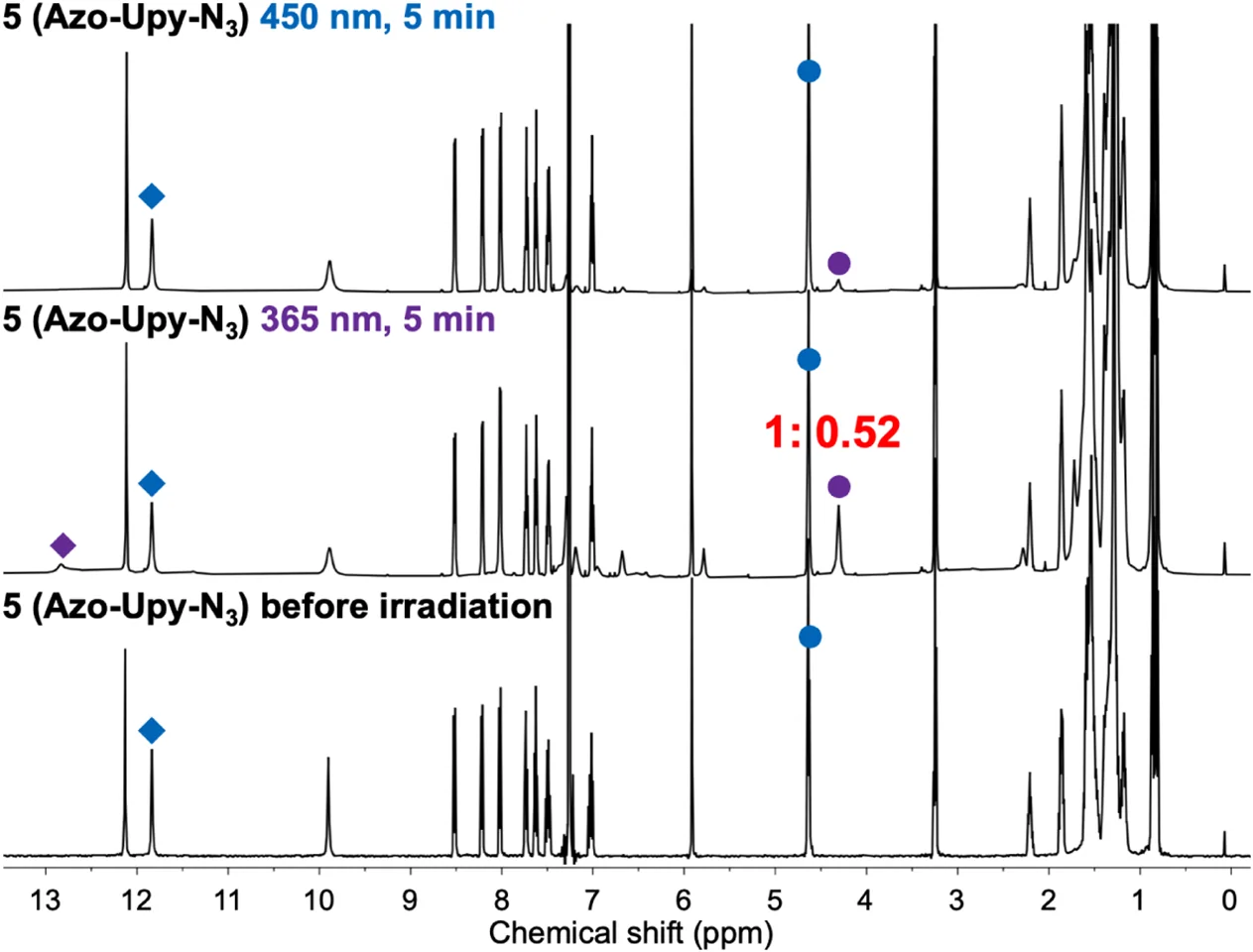
Stacked ^1^H NMR spectra of **5** (**Azo-Upy-N_3_**) before irradiation, after UV irradiation, and after subsequent blue light irradiation (600 MHz, 10.0 mM in CDCl_3_ at 25 °C).

### Postpolymerization Modification via CuAAC Click Chemistry

Having successfully synthesized clickable unit **5** and validated its photoresponsive dimerization behavior, we proceeded to graft it onto the helical PIC backbone via PPM using copper­(I)-catalyzed azide–alkyne cycloaddition (CuAAC) click chemistry (Figure 6). The alkyne-functionalized PIC backbone **P3** (**Alkyne-PIC**) was polymerized using the nickel-catalyzed system from the alkyne-functionalized isocyanide **M3** (**Alkyne-PhNC**). The PPM of **5** onto **P3** under CuAAC conditions was characterized by ^1^H NMR spectroscopy (Figure 7), in which the successful “clicking” of the Azo-Upy motif onto the PIC backbone is supported by the disappearance of the alkyne signals (δ ≈ 2.60 ppm in **M3** and **P3**, marked with red dots) in **P3P**, as well as the broadening signals of Azo-Upy motifs. This successful PPM is further corroborated by FT-IR spectroscopy (Figure S9), evidenced by the disappearance of the terminal alkyne stretching vibrations (∼3291 and 2128 cm^–1^), alongside the emergence of a broad hydrogen-bonded N–H stretching band (∼3265 cm^–1^), as well as the new signals within the 1650–1500 cm^–1^ region that can be attributed to the newly formed 1,2,3-triazole and aromatic rings. While **P3** shows good solubility in common organic solvents, **P3P** exhibits significantly limited solubility in THF or DMF, which precludes our standard characterization via GPC. The limited solubility, however, can be attributed to the successful incorporation of a high density of Azo-Upy motifs. We hypothesize that strong intermolecular aggregation, driven by quadruple hydrogen bonding and the rigidity of the PIC backbone, facilitates the formation of a stable supramolecular network in **P3P**, limiting its solubility.

**Figure 6:**
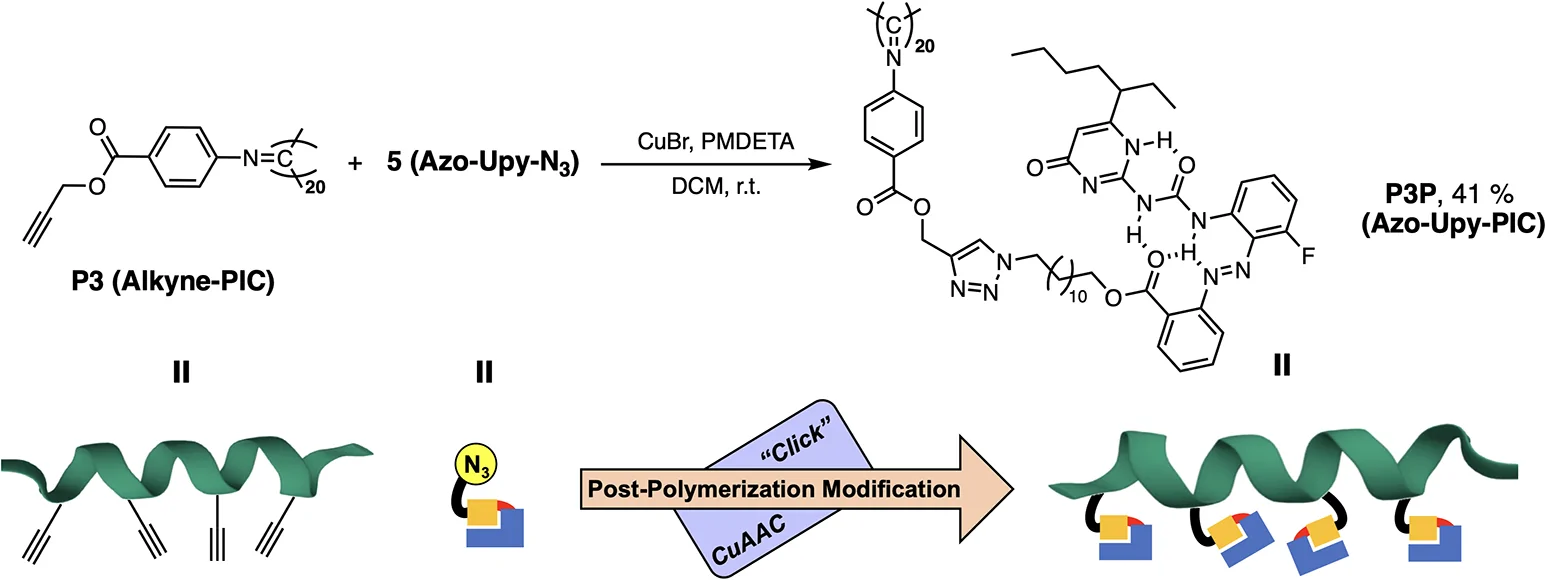
Postpolymerization modification of the PIC backbone **P3** with **5** (**Azo-Upy-N_3_**) via CuAAC click chemistry.

**Figure 7:**
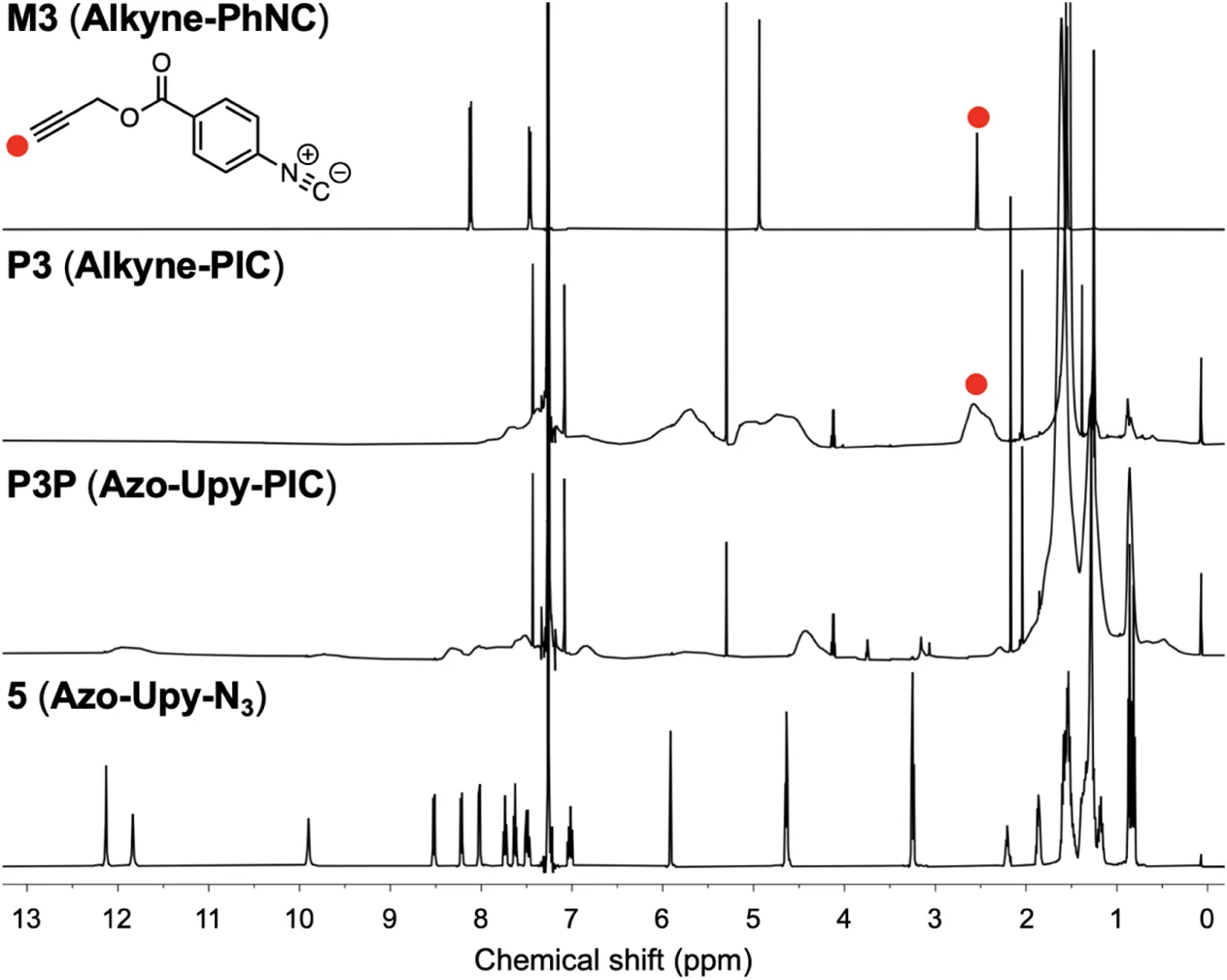
Stacked ^1^H NMR spectra illustrating the postpolymerization modification of **P3** (**Alkyne-PIC**) (600 MHz, 10.0 mM in CDCl_3_ at 25 °C).

## Conclusions

In summary, we have successfully synthesized side-chain functionalized helical PICs bearing photoswitchable Azo-Upy motifs. Through controlled nickel-catalyzed polymerization, well-defined PIC copolymers with a preferred helical handedness were accessed with low dispersities. These copolymers can undergo reversible quadruple hydrogen bonding upon UV/blue light irradiation. The assembly behavior can be steered between intrachain collapse and interchain cross-linking simply by tuning the polymer concentration or changing the solvent. Throughout these dynamic switching processes, we confirm the orthogonality between the dynamic side-chain interactions and the structural integrity of the static helical backbone. Significantly, to broaden the utility of the Azo-Upy motif for postpolymerization modification, we designed and synthesized a “clickable” **Azo-Upy-N**_**3**_ module. Beyond validating its reversible self-dimerization, 2D-DOSY NMR provides quantitative evidence for its volume expansion upon UV irradiation. We demonstrate that this **Azo-Upy-N**_**3**_ can be grafted onto the PIC backbone via CuAAC click chemistry, thereby bypassing the synthetic challenges associated with the sensitive isocyanide monomer. Collectively, this study establishes a foundational platform for realizing the controllable assembly of helical polymers into higher-order hierarchies, paving the way for engineering sophisticated, biomimetic materials where rigid scaffolds and dynamic supramolecular interactions can coexist and function cooperatively.

## Supplementary Material



## Notes

Detailed synthetic procedures and compound characterizations; IR spectra of nickel initiators and PPM product; DLS measurement results; optical characterizations of **5** (**Azo-Upy-N**_**3**_); 2D-DOSY NMR results; ^1^H-, ^13^C-, and ^19^F-NMR spectrum copies (PDF)
