# Supplementary material for: Photoresponsive Reversible Assembly of Helical Poly(isocyanide)s
Source: ACS Polym Au. 2026 Jul 31;6(5):1403–13. doi: 10.1021/acspolymersau.6c00119 (PMC13673882; doi:10.1021/acspolymersau.6c00119)
Supplement: Supplementary file 1 [file lg6c00119_si_001.pdf]

Supporting Information

Photo-Responsive Reversible Assembly of Helical Poly(isocyanide)s

Jingyang Wang, Federica Kauffmann, Chengyuan Wang, Matthew D. Hannigan, and Marcus Weck*

Molecular Design Institute and Department of Chemistry, New York University
New York, NY 10003 USA

*Email: marcus.weck@nyu.edu

Table of Contents

1. Synthetic Procedures and Compound Characterization ······ S2
   1.1 Synthesis and characterization of **M1** (**Azo-Upy-PhNC**) ······ S2
   1.2 Synthesis and characterization of **M2** (***L*-Men-PhNC**) ······ S3
   1.3 Synthesis and characterization of the nickel initiators ······ S3
   1.4 Synthesis and characterization of the clickable unit **5** (**Azo-Upy-N_3**) ······ S4
   1.5 Synthesis and characterization of **M3** (**Alkyne-PhNC**) ······ S6
2. Comparison of the Photo-Responsiveness of **P1a**, **P1b**, and **P1c** ······ S7
3. DLS Measurement Results of **P1a** ······ S7
4. IR Characterization of the Post-Polymerization Modification ······ S9
5. Characterization of the Self-Dimerization of **5** (**Azo-Upy-N_3**) ······ S11
References ······ S12
^{1}H-, ^{13}C-, and ^{19}F- NMR Spectra ······ S13

1. Synthetic Procedures and Compound Characterization

1.1 Synthesis and characterization of **M1** (**Azo-Upy-PhNC**)

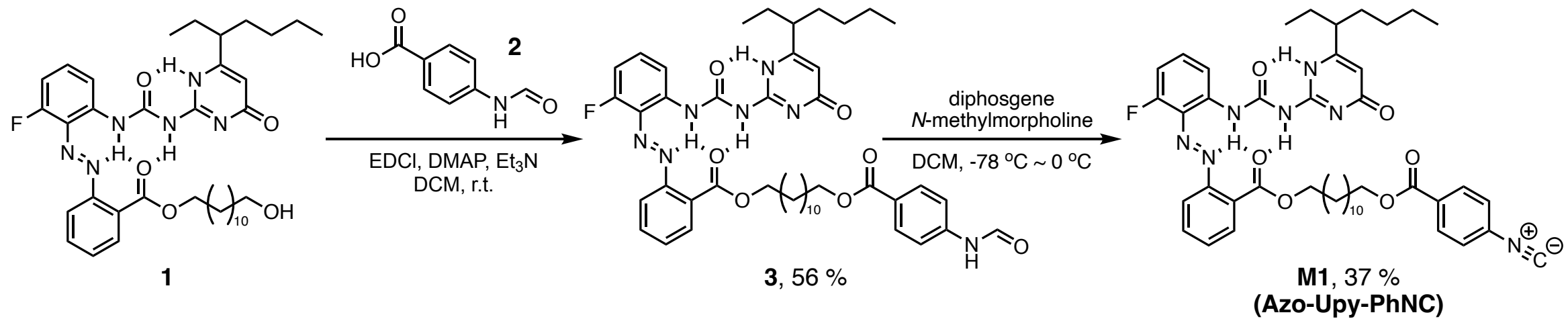

Compounds 1[1] and **2**[2] were prepared and characterized according to literature-reported procedures.

Compound 3

Into a stirring solution of compound **1** (1.00 g, 1.47 mmol) in DCM (25 mL) were added DMAP (45.0 mg, 0.368 mmol), Et_3N (149 mg, 1.47 mmol), compound **2** (364 mg, 2.21 mmol), and EDCI (704 mg, 3.68 mmol). The resulting red slurry was stirred at room temperature until the system became clear red and TLC monitoring suggested full consumption of compound **1**. Then, the reaction mixture was diluted with DCM (30 mL), continuously washed with a saturated aqueous solution of sodium bicarbonate (40 mL), 1.0 M HCl aqueous solution (40 mL), and brine (40 mL), respectively. The organic phase was dried over anhydrous Na_2SO_4, filtrated, and concentrated *in vacuo*. The crude product was further purified by flash column chromatography on silica with a ternary eluent of hexanes/EtOAc/MeOH = 20/10/1, obtaining the pure compound **3** as an orange to red solid (681 mg, 56 %). ^{1}H NMR (500 MHz, $CDCl_3$, 298 K) δ 12.12 (d, *J* = 2.6 Hz, 1H), 11.87 (d, *J* = 16.7 Hz, 1H), 9.91 (s, 1H), 8.51 (d, *J* = 8.6 Hz, 1H), 8.45 (d, *J* = 1.7 Hz, 1H), 8.21 (dd, *J* = 7.9, 1.5 Hz, 1H), 8.07 – 7.98 (m, 3H), 7.94 (s, 1H), 7.76 – 7.69 (m, 1H), 7.68 – 7.59 (m, 2H), 7.49 (td, *J* = 8.5, 6.0 Hz, 1H), 7.11 (d, *J* = 8.3 Hz, 1H), 7.04 – 6.97 (m, 1H), 5.91 (d, *J* = 2.5 Hz, 1H), 4.63 (t, *J* = 6.0 Hz, 2H), 4.29 (q, *J* = 6.1 Hz, 2H), 2.25 – 2.16 (m, 1H), 1.86 (dq, *J* = 12.1, 6.3 Hz, 2H), 1.80 – 1.68 (m, 3H), 1.52 (dqd, *J* = 16.0, 7.2, 4.8 Hz, 6H), 1.41 (dq, *J* = 13.8, 6.9 Hz, 5H), 1.36 – 1.22 (m, 9H), 1.16 (tt, *J* = 8.6, 6.1 Hz, 3H), 0.87 – 0.83 (m, 3H), 0.81 (td, *J* = 7.4, 3.8 Hz, 3H). ^{13}C NMR (126 MHz, $CDCl_3$, 298 K) δ 172.2, 167.2, 166.1, 163.0, 162.2, 161.3, 161.0, 159.1, 153.9, 151.2, 151.0, 141.1, 134.74, 134.66, 134.2, 131.7, 131.6, 131.5, 131.4, 130.8, 128.7, 126.4, 126.1, 119.1, 117.2, 116.3, 115.8, 110.4, 110.3, 107.5, 107.4, 67.1, 65.2, 65.1, 49.2, 31.6, 29.7, 29.58, 29.55, 29.52, 29.50, 29.45, 29.4, 29.33, 29.31, 28.8, 28.7, 27.0, 26.4, 26.12, 26.05, 22.8, 14.1, 12.0. ^{19}F NMR (471 MHz, $CDCl_3$, 298 K) δ -117.41, -117.43. LC-TOF (ESI) Calcd. For $C_{45}H_{56}FN_7O_7$ $[M+H]^+$ *m/z* 826.4298, Found: 826.4249.

M1 (**Azo-Upy-PhNC**)

A solution of compound **3** (500 mg, 0.605 mmol) and *N*-methylmorpholine (245 mg, 2.42 mmol) in dry DCM (40 mL) was cooled to -78 ºC, to which a solution of diphosgene (108 mg, 0.545 mmol) in dry DCM (5.0 mL) was added dropwise using a syringe under a N_2 atmosphere. After the addition, the reaction mixture was stirred at -78 ºC for 5 minutes,

before it was allowed to warm to 0 °C and was stirred for another 10 minutes. Then, a saturated aqueous solution of sodium bicarbonate (25 mL, 0 °C) was added to quench the dehydration, and the resulting mixture was stirred vigorously for 15 minutes. After the extraction, the system was washed with a saturated aqueous solution of sodium bicarbonate (25 mL × 2), followed by brine (25 mL). The organic phase was dried over anhydrous Na_2SO_4, filtrated, and concentrated *in vacuo*. The crude product was further purified by flash column chromatography on silica with a binary eluent of hexanes/EtOAc = 3/1, obtaining the pure **M1** (**Azo-Upy-PhNC**) as an orange solid (180 mg, 37 %). ^{1}H NMR (500 MHz, $CDCl_3$, 298 K) δ 12.11 (s, 1H), 11.83 (s, 1H), 9.86 (s, 1H), 8.51 (d, *J* = 8.7 Hz, 1H), 8.21 (d, *J* = 7.9 Hz, 1H), 8.07 (d, *J* = 8.2 Hz, 2H), 8.01 (d, *J* = 8.1 Hz, 1H), 7.73 (t, *J* = 7.7 Hz, 1H), 7.62 (t, *J* = 7.6 Hz, 1H), 7.49 (td, *J* = 8.4, 5.9 Hz, 1H), 7.44 (d, *J* = 8.2 Hz, 2H), 7.01 (t, *J* = 9.1 Hz, 1H), 5.91 (s, 1H), 4.63 (t, *J* = 6.0 Hz, 2H), 4.32 (t, *J* = 6.7 Hz, 2H), 2.20 (dt, *J* = 9.0, 4.2 Hz, 1H), 1.86 (dq, *J* = 12.0, 6.3 Hz, 2H), 1.75 (p, *J* = 6.9 Hz, 2H), 1.54 (tq, *J* = 18.1, 10.5, 8.4 Hz, 6H), 1.40 (dq, *J* = 11.5, 6.1, 5.1 Hz, 6H), 1.36 – 1.22 (m, 12H), 0.86 (t, *J* = 7.4 Hz, 3H), 0.82 (t, *J* = 7.4 Hz, 3H). ^{13}C NMR (126 MHz, $CDCl_3$, 298 K) δ 172.0, 167.3, 165.2, 162.2, 161.1, 154.0, 151.4, 151.1, 134.9, 134.8, 134.4, 131.9, 131.7, 131.5, 131.4, 130.9, 128.9, 128.8, 126.6, 126.3, 116.5, 116.0, 110.5, 110.4, 107.7, 67.3, 65.9, 49.3, 33.7, 32.1, 29.9, 29.8, 29.73, 29.71, 29.68, 29.5, 29.4, 28.82, 28.77, 27.1, 26.6, 26.1, 23.0, 14.2, 12.1. ^{19}F NMR (471 MHz, $CDCl_3$, 298 K) δ -117.44. LC-TOF (ESI) Calcd. For $C_{45}H_{54}FN_7O_6$ $[M+H]^+$ *m/z* 808.4192, Found: 808.4176.

1.2 Synthesis and characterization of **M2** (***L*-Men-PhNC**)

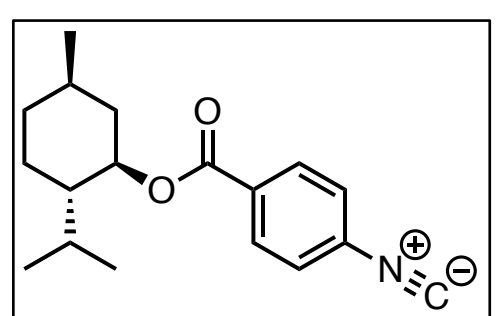

M2 (*L*-Men-PhNC)

M2 (***L*-Men-PhNC**) was prepared and characterized according to a literature-reported procedure[3] as a pale green solid.

1.3 Synthesis and characterization of the nickel initiators

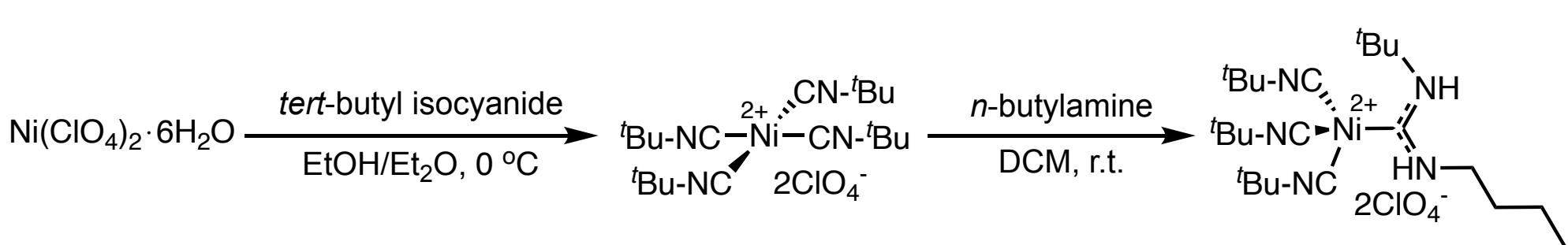

I1, 85 %   **I2**, 97 %

I1

In a 50 mL round bottom flask, nickel (II) perchlorate hexahydrate (808 mg, 2.21 mmol) was dissolved in $EtOH/Et_2O$ (12.0 mL, *v*/*v*, 50/50). The resulting greenish solution was cooled to 0 °C in an ice bath. Then, *tert*-butyl isocyanide (735 mg, 8.84 mmol) was added dropwise as the color of the mixture flashed red/orange, then returned to green until all equivalents were added and the mixture finally turned into an opaque pale-orange slurry. The product was collected by vacuum filtration and washed first with cold $EtOH/Et_2O$ (20

mL) and then with cold Et_2O (20 mL). The pale-yellow solid was dried *in vacuo* until the solid became white (1.10 g, 85 %). The yielded **I1** was used for polymerization without further purification.

I2

In a 25 mL oven-dried round bottom flask, **I1** (133 mg, 0.224 mmol) was dissolved in dry, degassed DCM (6.0 mL) under an argon atmosphere. To the resulting yellow slurry was added *n*-butylamine (17.8 mg, 0.243 mmol) dissolved in dry, degassed DCM (1.5 mL). The reaction mixture turned into an orange solution as it was stirred for another 15 minutes at room temperature. After adding hexanes (5.0 mL), the product was then precipitated out and collected by vacuum filtration as an orange solid (144 mg, 97 %). The yielded **I2** was used for polymerization without further purification.

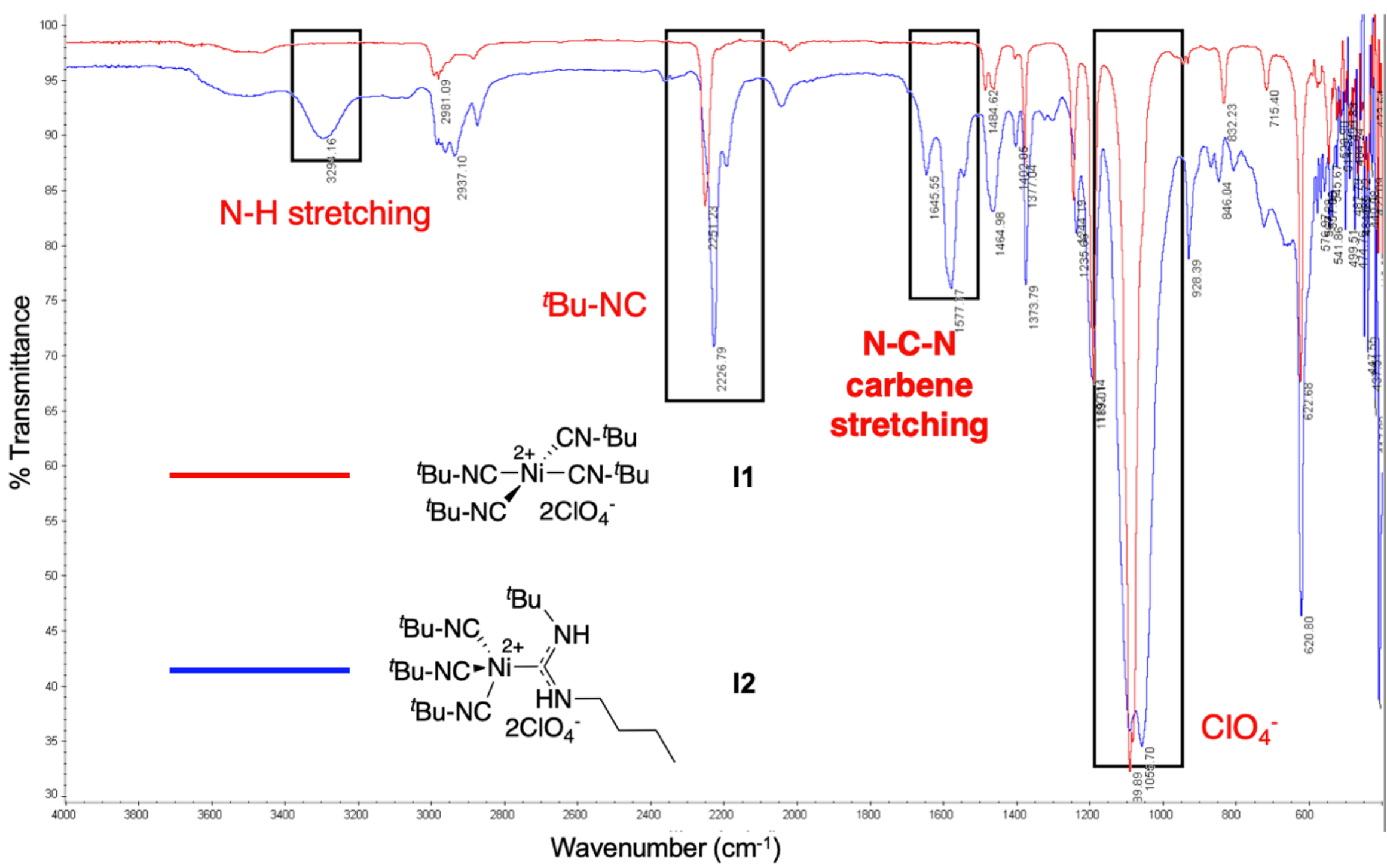

Figure S1. IR spectra of nickel initiators **I1** and **I2**.

1.4 Synthesis and characterization of the clickable unit **5** (**Azo-Upy-N$_3$**)

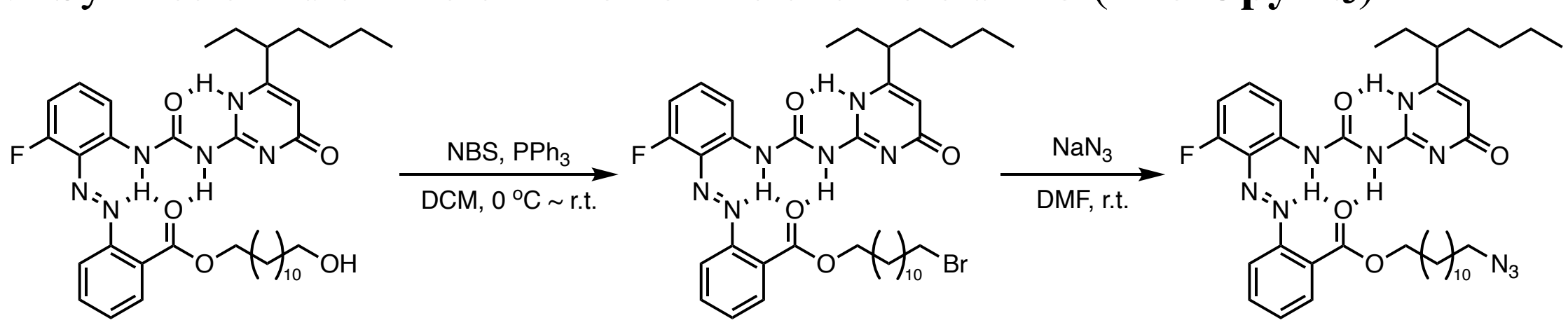

1 **4**, 82 % **5**, 90 % **(Azo-Upy-N$_3$)**

Compound 1 was prepared according to a literature-reported procedure.[1]

Compound 4

Into a stirring solution of PPh_3 (965 mg, 3.69 mmol) in DCM (40 mL) was added NBS (655 mg, 3.69 mmol). The mixture was cooled to 0 °C and stirred for 10 minutes to which compound **1** (1.00 g, 1.47 mmol) was then added in one portion. The reaction mixture was stirred at 0 °C for 15 minutes, before it was allowed to warm to room temperature and was stirred for another 2 hours, resulting in a dark brown solution. Without further work-up steps, the reaction mixture was concentrated *in vacuo*. The crude product was further purified by flash column chromatography on silica with a binary eluent of hexanes/EtOAc = 3/1, obtaining the pure compound **4** as an orange solid (894 mg, 82 %). ^{1}H NMR (500 MHz, $CDCl_3$, 298 K) δ 12.13 (s, 1H), 11.85 (s, 1H), 9.94 (s, 1H), 8.51 (d, *J* = 8.7 Hz, 1H), 8.21 (dd, *J* = 7.8, 1.6 Hz, 1H), 8.02 (dd, *J* = 8.2, 1.3 Hz, 1H), 7.77 – 7.70 (m, 1H), 7.63 (td, *J* = 7.6, 1.3 Hz, 1H), 7.49 (td, *J* = 8.5, 6.0 Hz, 1H), 7.02 (t, *J* = 9.1 Hz, 1H), 5.92 (s, 1H), 4.63 (t, *J* = 6.0 Hz, 2H), 3.40 (t, *J* = 6.8 Hz, 2H), 2.21 (d, *J* = 4.9 Hz, 1H), 1.90 – 1.79 (m, 4H), 1.56 (m, 10H), 1.40 (dq, *J* = 11.3, 6.0 Hz, 4H), 1.35 – 1.24 (m, 12H), 1.22 – 1.13 (m, 2H), 0.86 (t, *J* = 7.2 Hz, 3H), 0.82 (t, *J* = 7.4 Hz, 3H). ^{13}C NMR (151 MHz, $CDCl_3$, 298 K) δ 172.0, 167.3, 163.0, 162.1, 161.3, 154.0, 151.3, 151.1, 134.8, 134.7, 134.3, 131.8, 131.7, 131.4, 128.9, 128.8, 126.2, 116.5, 115.92, 115.89, 110.5, 110.3, 107.7, 67.3, 49.3, 34.1, 33.7, 32.9, 29.8, 29.68, 29.66, 29.64, 29.56, 29.5, 28.9, 28.8, 28.3, 27.1, 26.5, 22.9, 14.2, 12.1. ^{19}F NMR (471 MHz, $CDCl_3$, 298 K) δ -117.48. LC-TOF (ESI) Calcd. For $C_{37}H_{50}BrFN_6O_4$ $[M+H]^+$ *m/z* 741.3134, Found: 741.3082.

Compound 5 (**Azo-Upy-N_3**)

Into a stirring solution of compound **4** (500 mg, 0.674 mmol) in dry DMF (25 mL) was added sodium azide (48.2 mg, 0.741 mmol) in one portion. The resulting red solution was stirred at room temperature for 16 hours until TLC monitoring suggested full consumption of compound **4**. Then, the reaction mixture was extracted with water (150 mL) and ethyl acetate (100 mL). The organic phase was continuously washed with brine (80 mL), dried over anhydrous Na_2SO_4, filtrated, and concentrated *in vacuo*. The crude product was further purified by flash column chromatography on silica with a binary eluent of hexanes/EtOAc = 3/1, obtaining the pure compound **5** (**Azo-Upy-N_3**) as an orange solid (427 mg, 90 %). ^{1}H NMR (500 MHz, $CDCl_3$, 298 K) δ 12.13 (s, 1H), 11.84 (s, 1H), 9.90 (s, 1H), 8.52 (d, *J* = 8.7 Hz, 1H), 8.22 (dd, *J* = 7.8, 1.5 Hz, 1H), 8.02 (dd, *J* = 8.2, 1.2 Hz, 1H), 7.74 (td, *J* = 7.8, 1.5 Hz, 1H), 7.63 (td, *J* = 7.6, 1.3 Hz, 1H), 7.50 (td, *J* = 8.5, 6.1 Hz, 1H), 7.01 (dd, *J* = 10.0, 8.2 Hz, 1H), 5.91 (d, *J* = 1.5 Hz, 1H), 4.64 (t, *J* = 6.0 Hz, 2H), 3.25 (t, *J* = 6.9 Hz, 2H), 2.20 (tq, *J* = 9.8, 4.8 Hz, 1H), 1.86 (dt, *J* = 12.0, 6.2 Hz, 2H), 1.63 – 1.44 (m, 12H), 1.42 – 1.22 (m, 16H), 1.18 (q, *J* = 7.4 Hz, 2H), 0.86 (t, *J* = 7.2 Hz, 3H), 0.82 (t, *J* = 7.4 Hz, 3H). ^{13}C NMR (151 MHz, $CDCl_3$, 298 K) δ 172.0, 167.3, 163.0, 162.1, 161.3, 154.0, 151.4, 151.1, 134.83, 134.76, 134.3, 131.9, 131.7, 131.5, 128.90, 128.86, 126.3, 116.5, 116.0, 115.9, 110.5, 110.4, 107.7, 67.3, 53.6, 51.6, 49.3, 33.7, 29.8, 29.70, 29.68, 29.66, 29.6, 29.5, 29.3, 29.0, 28.8, 27.1, 26.9, 26.6, 23.0, 14.2, 12.1. ^{19}F NMR (471 MHz, $CDCl_3$, 298 K) δ -117.48. LC-TOF (ESI) Calcd. For $C_{37}H_{50}FN_9O_4$ $[M+H]^+$ *m/z* 704.4043, Found: 704.4105.

1.5 Synthesis and characterization of **M3** (**Alkyne-PhNC**)

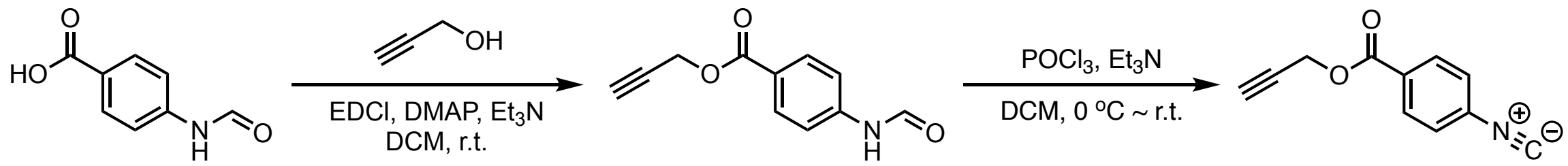

2 **6**, 81 % **M3**, 92 % **(Alkyne-PhNC)**

Compounds 2 was prepared and characterized according to a literature-reported procedure.[2]

Compound 6

Into a stirring suspension of compound **2** (200 mg, 1.21 mmol) in DCM (20 mL) were added DMAP (14.8 mg, 0.121 mmol), Et_3N (123 mg, 1.21 mmol), and EDCI (580 mg, 3.03 mmol). The mixture was stirred at room temperature for 10 minutes, after which prop-2-yn-1-ol (74.7 mg, 1.33 mmol) in DCM (10 mL) was added dropwise. The resulting slightly yellow slurry was then stirred at room temperature for 16 hours until the system became clear yellow solution. Then, the reaction mixture was diluted with DCM (30 mL), continuously washed with a saturated aqueous solution of sodium bicarbonate (40 mL), 1.0 M HCl aqueous solution (40 mL), and brine (40 mL), respectively. The organic phase was dried over anhydrous Na_2SO_4, filtrated, and concentrated *in vacuo*. The yellow crude product was further purified by flash column chromatography on silica with a binary eluent of DCM/EtOAc = 4/1, obtaining compound **6** as an off-white solid (200 mg, 81 %). ^1H NMR (600 MHz, Acetone-d_6, 298 K) δ 8.45 (d, J = 1.3 Hz, 1H), 8.01 (dd, J = 9.1, 2.4 Hz, 2H), 7.86 – 7.79 (m, 2H), 4.95 (d, J = 2.5 Hz, 2H), 3.08 (t, J = 2.5 Hz, 1H), 2.79 (d, J = 20.2 Hz, 1H). ^{13}C NMR (151 MHz, Acetone-d_6, 298 K) δ 165.7, 160.6, 144.0, 132.3, 131.7, 125.6, 119.8, 117.7, 79.2, 76.6, 52.9. LC-TOF (ESI) Calcd. For $C_{11}H_9NO_3$ $[M+H]^+$ *m/z* 204.0655, Found: 204.0660.

M3 (**Alkyne-PhNC**)

Into a stirring solution of compound **6** (180 mg, 0.885 mmol) and Et_3N (448 mg, 4.43 mmol) in DCM (20 mL) was added $POCl_3$ (204 mg, 1.33 mmol) in DCM (2.0 mL) dropwise at 0 ºC in an ice bath. The resulting clear light-yellow solution was stirred at 0 ºC for 10 minutes, before it was allowed to warm up to room temperature and was stirred for another 16 hours. Then, the reaction mixture was diluted with DCM (25 mL), continuously washed with a saturated aqueous solution of sodium bicarbonate (30 mL × 2), and brine (40 mL), respectively. The organic phase was dried over anhydrous Na_2SO_4, filtrated, and concentrated *in vacuo*. The crude product as a brown sludge was further purified by flash column chromatography on neutral alumina with a binary eluent of hexanes/EtOAc = 10/1, obtaining **M3** (**Alkyne-PhNC**) as a white solid (151 mg, 92 %). ^1H NMR (600 MHz, $CDCl_3$, 298 K) δ 8.13 – 8.09 (m, 2H), 7.46 (d, J = 8.6 Hz, 2H), 4.94 (d, J = 2.5 Hz, 2H), 2.54 (t, J = 2.5 Hz, 1H). ^{13}C NMR (151 MHz, $CDCl_3$, 298 K) δ 167.6, 164.4, 131.2, 130.4, 126.7, 77.3, 75.6, 53.1. LC-TOF (ESI) Calcd. For $C_{11}H_7NO_2$ $[M+H]^+$ *m/z* 186.0550, Found: 186.0526.

2. Comparison of the Photo-Responsiveness of P1a, P1b, and P1c

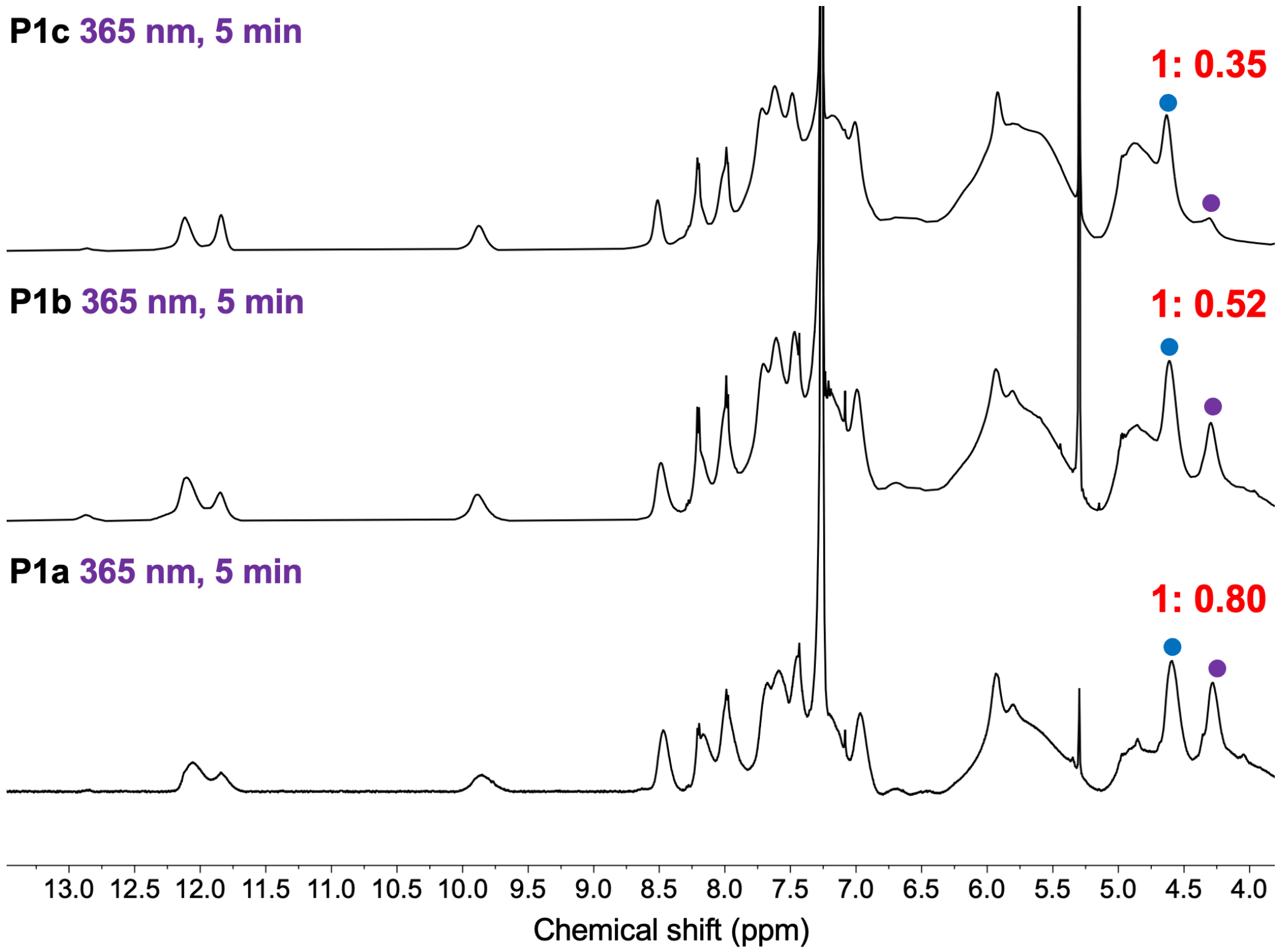

Figure S2. Stacked ^{1}H-NMR spectra of **P1a**, **P1b**, and **P1c** after UV irradiation. (600 MHz, 7.5 mg/mL of each copolymer sample in $CDCl_3$ at 25 °C)

3. DLS Measurement Results of P1a

Table S1. Hydrodynamic diameters of **P1a** measured by DLS (size distributions by volume).

**Sample**	**Solvent**	**Conc. (mg/mL)**	D_h **(nm)**
**P1a** before irradiation	$CHCl_3$	1.0	6.9 ± 1.3
**P1a** 365 nm, 5 min	$CHCl_3$	1.0	6.3 ± 1.4
**P1a** 450 nm, 5 min	$CHCl_3$	1.0	6.8 ± 1.4
**P1a** before irradiation	$CHCl_3$	10.0	6.5 ± 1.9
**P1a** 365 nm, 5 min	$CHCl_3$	10.0	7.6 ± 2.6
**P1a** 450 nm, 5 min	$CHCl_3$	10.0	6.6 ± 2.0

**P1a** before irradiation	THF	1.0	6.3 ± 2.0
**P1a** 365 nm, 5 min	THF	1.0	7.1 ± 2.0
**P1a** 450 nm, 5 min	THF	1.0	6.4 ± 1.8
**P1a** before irradiation	THF	10.0	7.7 ± 2.0
**P1a** 365 nm, 5 min	THF	10.0	8.6 ± 2.3
**P1a** 450 nm, 5 min	THF	10.0	7.8 ± 2.2

(D_h measured at 25 °C)

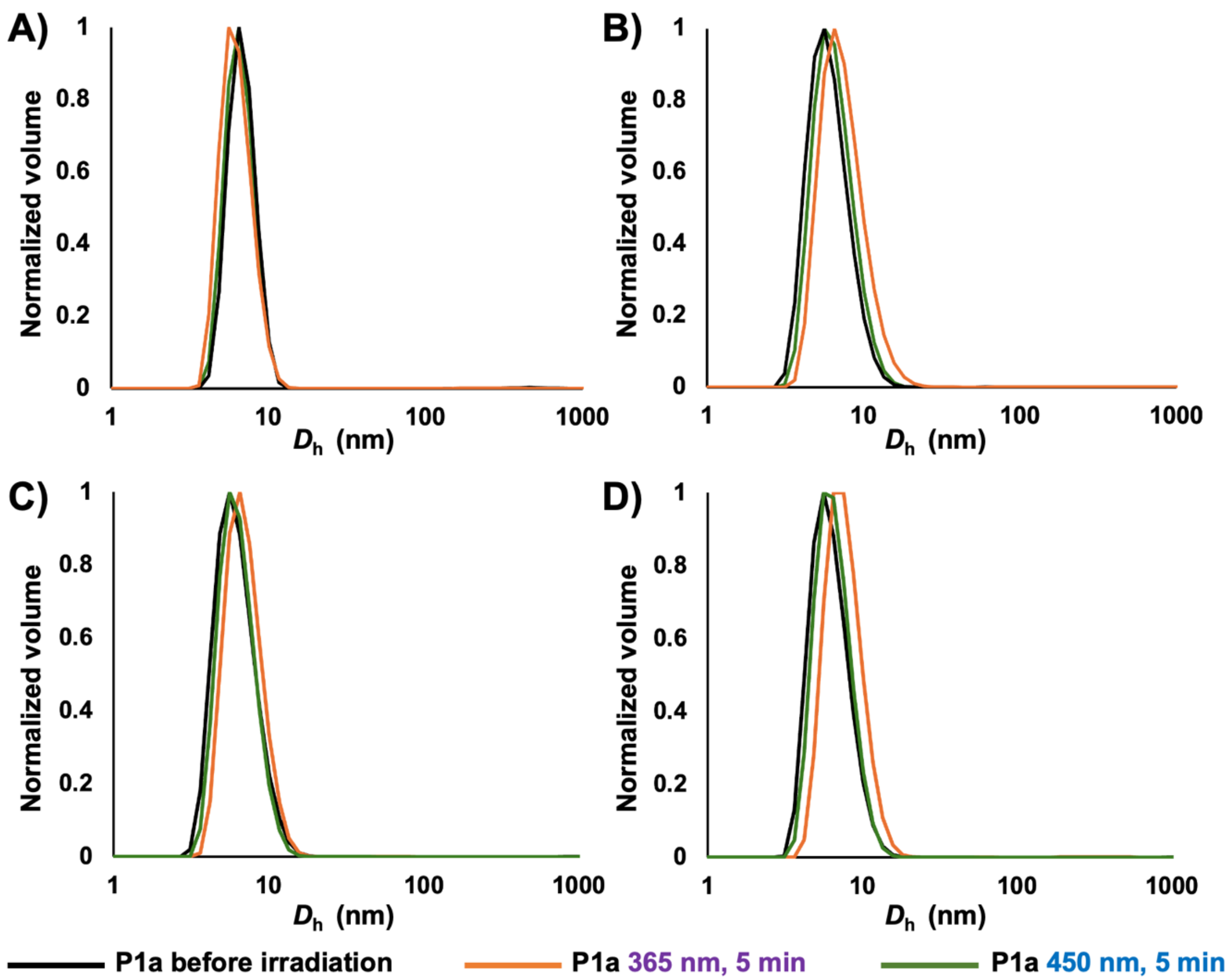

Figure S3. DLS profiles (size distributions by volume) of **P1a** before irradiation, after UV irradiation, and after subsequent blue light irradiation at (A) 1.0 mg/mL in $CHCl_3$, (B) 10.0 mg/mL in $CHCl_3$, (C) 1.0 mg/mL in THF, and (D) 10.0 mg/mL in THF at 25 °C.

4. Characterization of the Self-Dimerization of 5 (Azo-Upy-N$_3$)

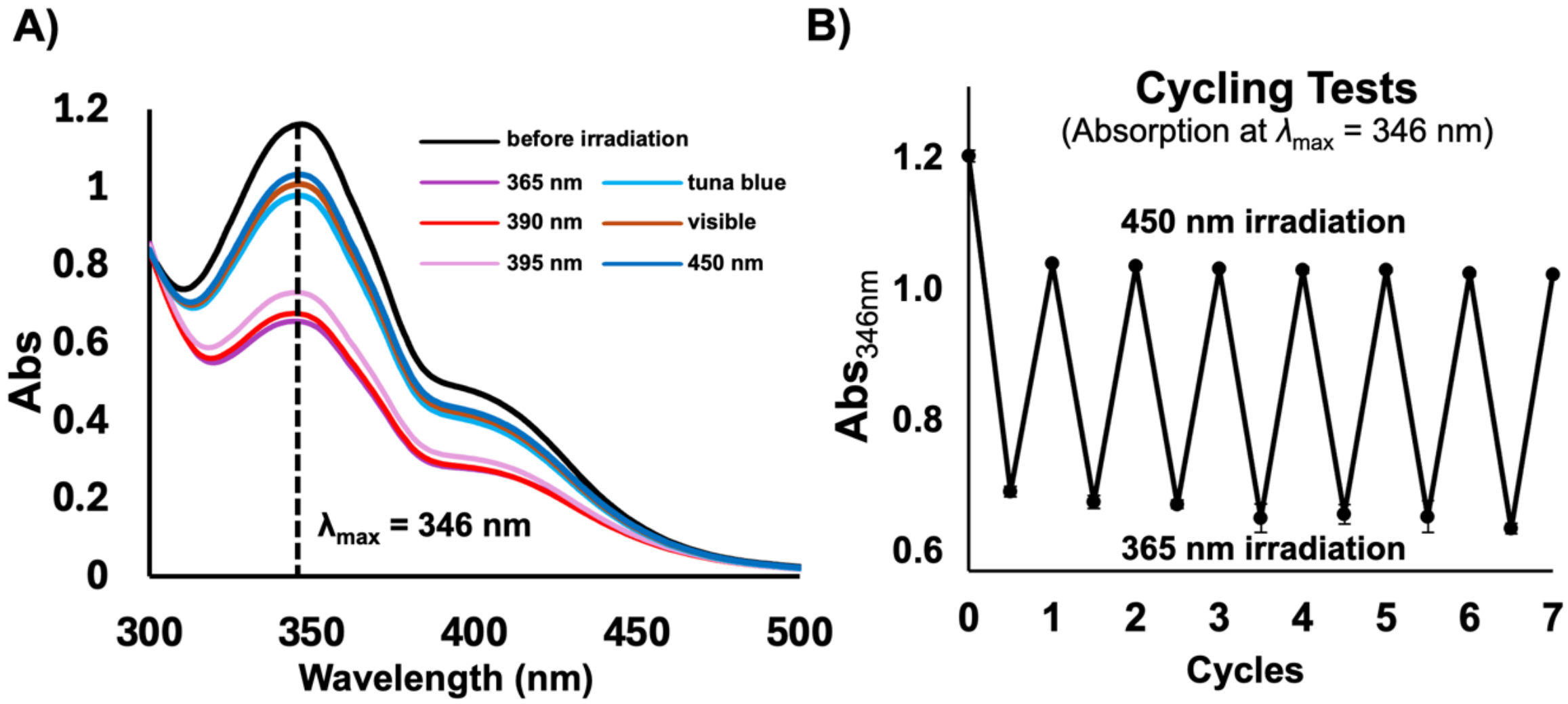

Figure S4. (A) UV-Vis spectra of **5** (**Azo-Upy-N$_3$**) before irradiation and after irradiation with different wavelengths. (B) Plot of absorption at λ_{max} = 346 nm after each irradiation (λ = 365 nm or 450 nm) of **5** (**Azo-Upy-N$_3$**) (0.10 mM in $CHCl_3$ at 25 °C, l = 1.0 cm, irradiation time: 5 min.).

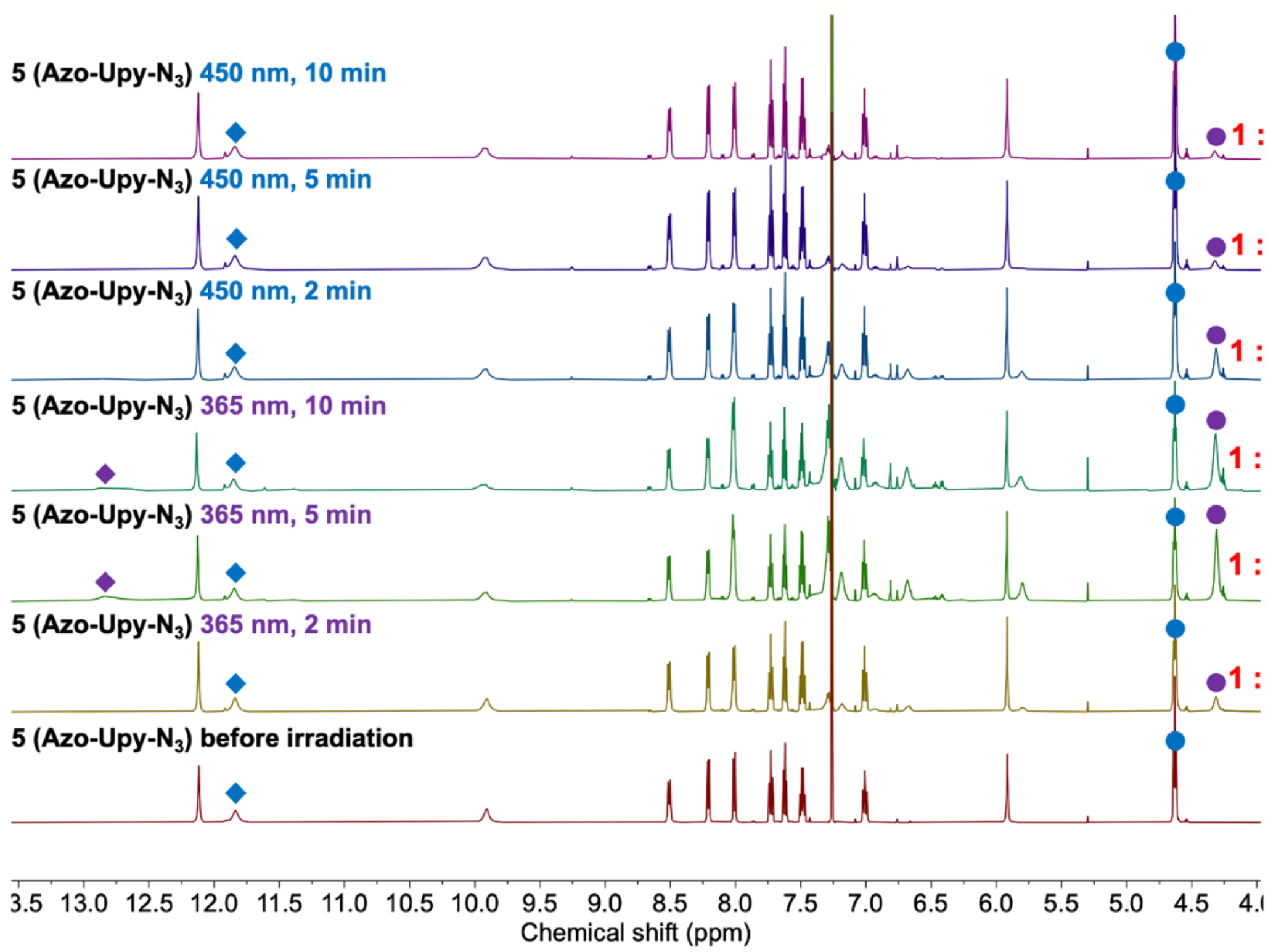

Figure S5. Time-dependent ^{1}H-NMR spectra of **5** (**Azo-Upy-N$_3$**) before irradiation, after UV irradiation, and after subsequent blue light irradiation (600 MHz, 10.0 mM in $CDCl_3$ at 25 °C).

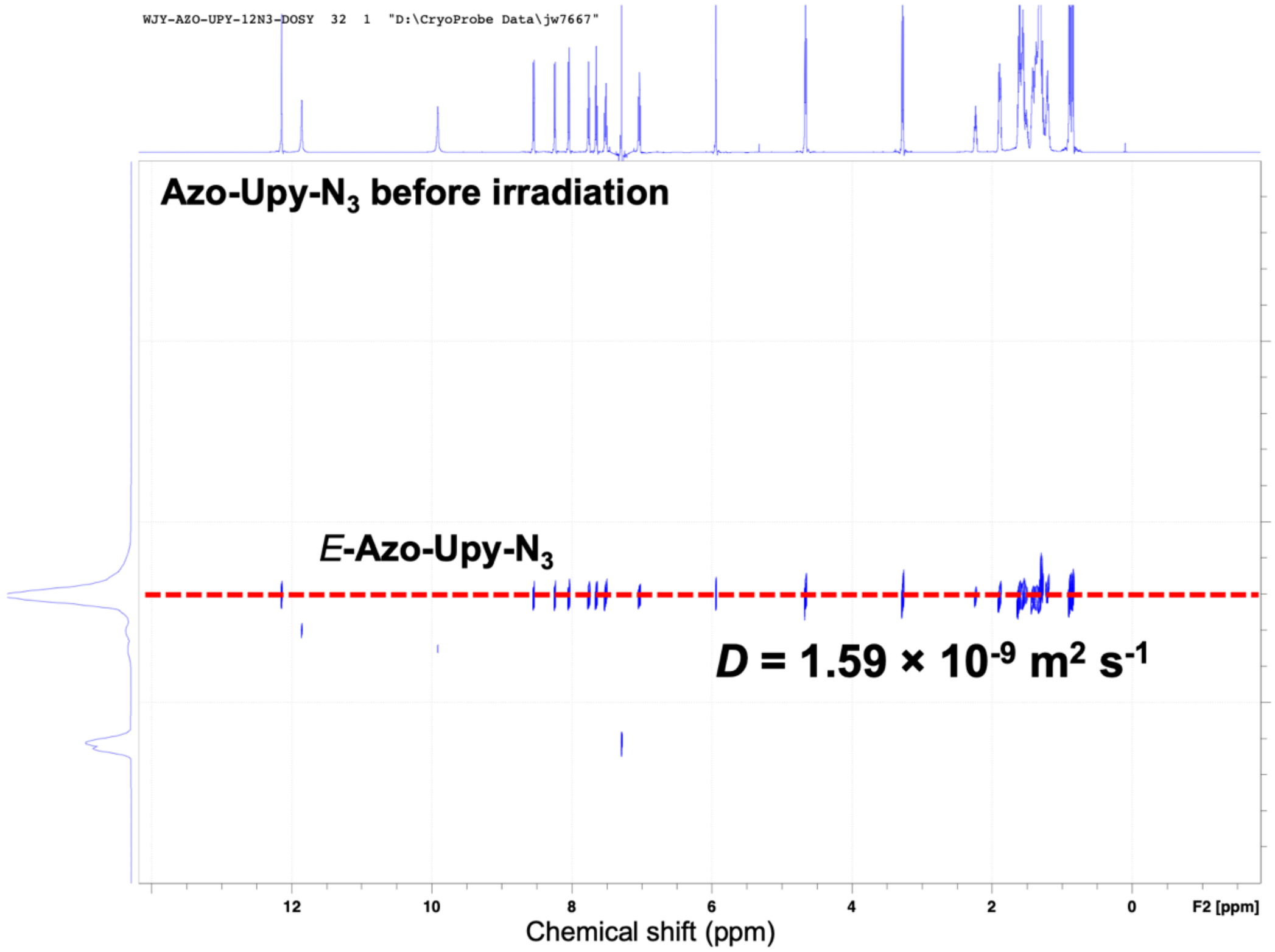

Figure S6. 2D-DOSY NMR spectrum of **5** (**Azo-Upy-N$_3$**) before irradiation (600 MHz, 10.0 mM in $CDCl_3$ at 25 ºC).

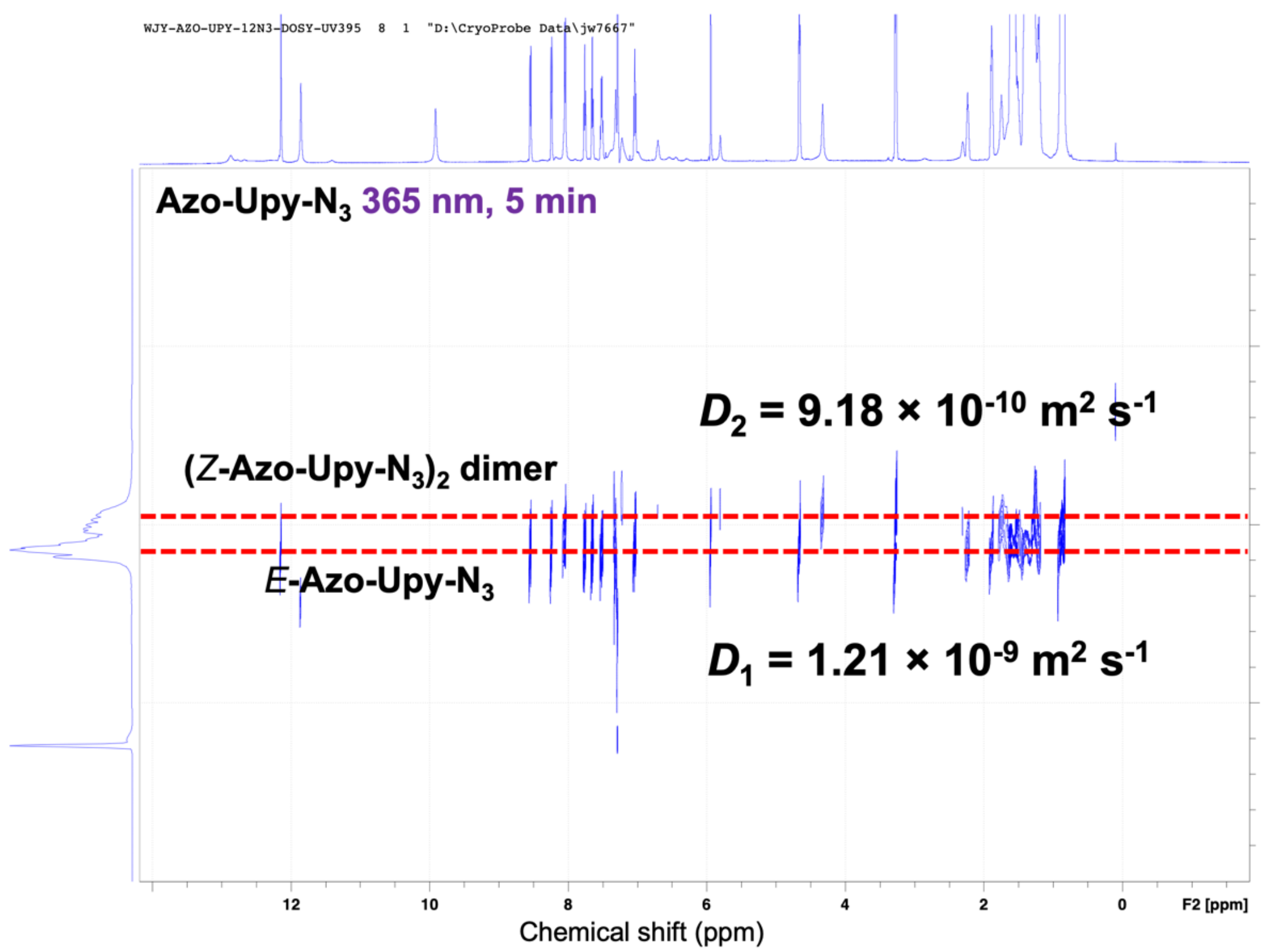

Figure S7. 2D-DOSY NMR spectrum of **5** (**Azo-Upy-N$_3$**) after UV irradiation (600 MHz, 10.0 mM in $CDCl_3$ at 25 ºC).

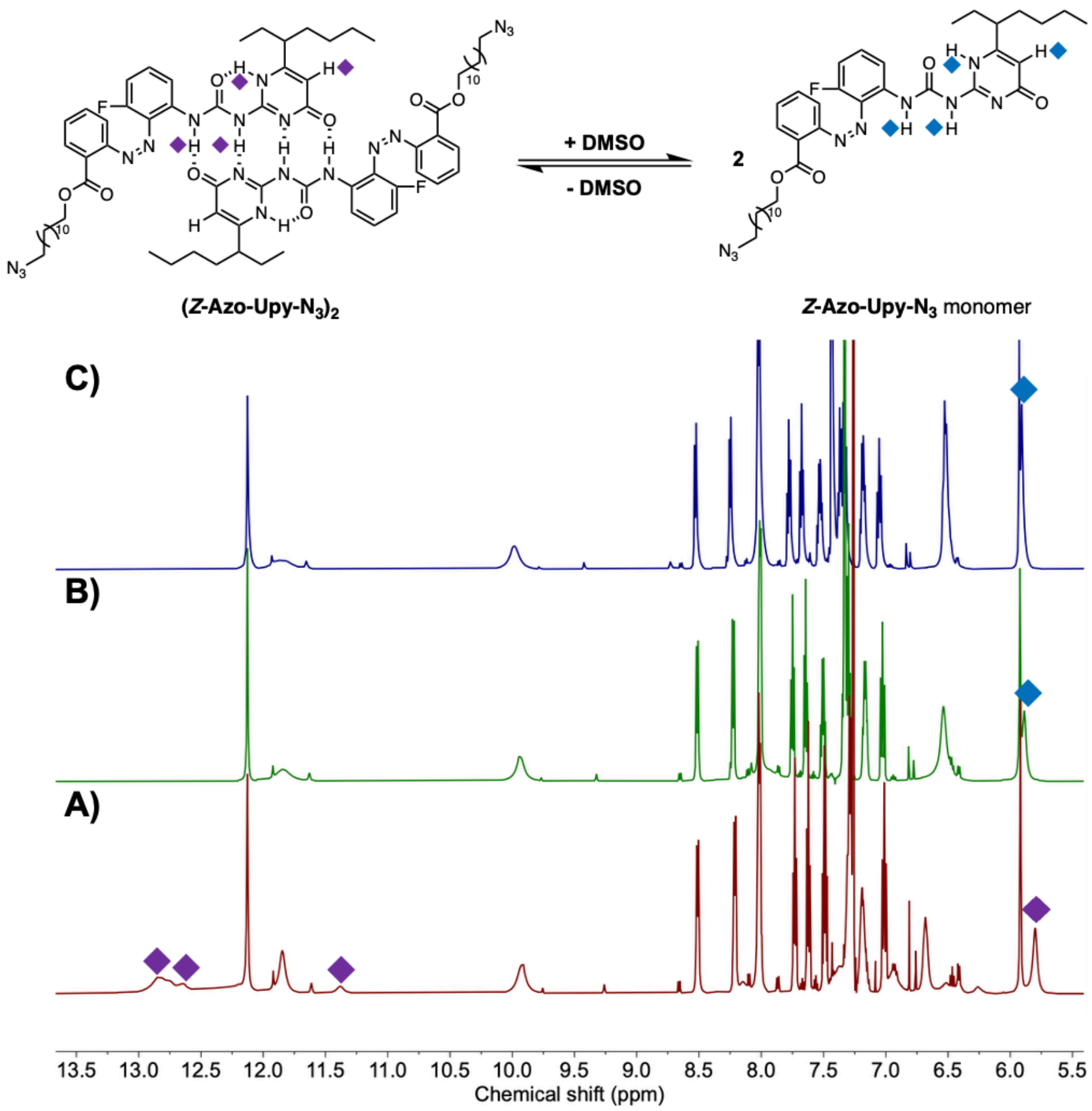

Figure S8. Schematic representation of the disassociation behavior of (***Z*-Azo-Upy-N$_3$**)$_2$ dimer upon addition of DMSO, and the ^{1}H-NMR spectra of the UV-irradiated solution of **5** (**Azo-Upy-N$_3$**) in A) $CDCl_3$, B) $CDCl_3$/DMSO-d_6 (v/v, 19/1), C) $CDCl_3$/DMSO-d_6 (v/v, 9/1). (600 MHz, 10.0 mM, 298 K; λ = 365 nm, irradiation time: 5 min)

5. IR Characterization of the Post-Polymerization Modification

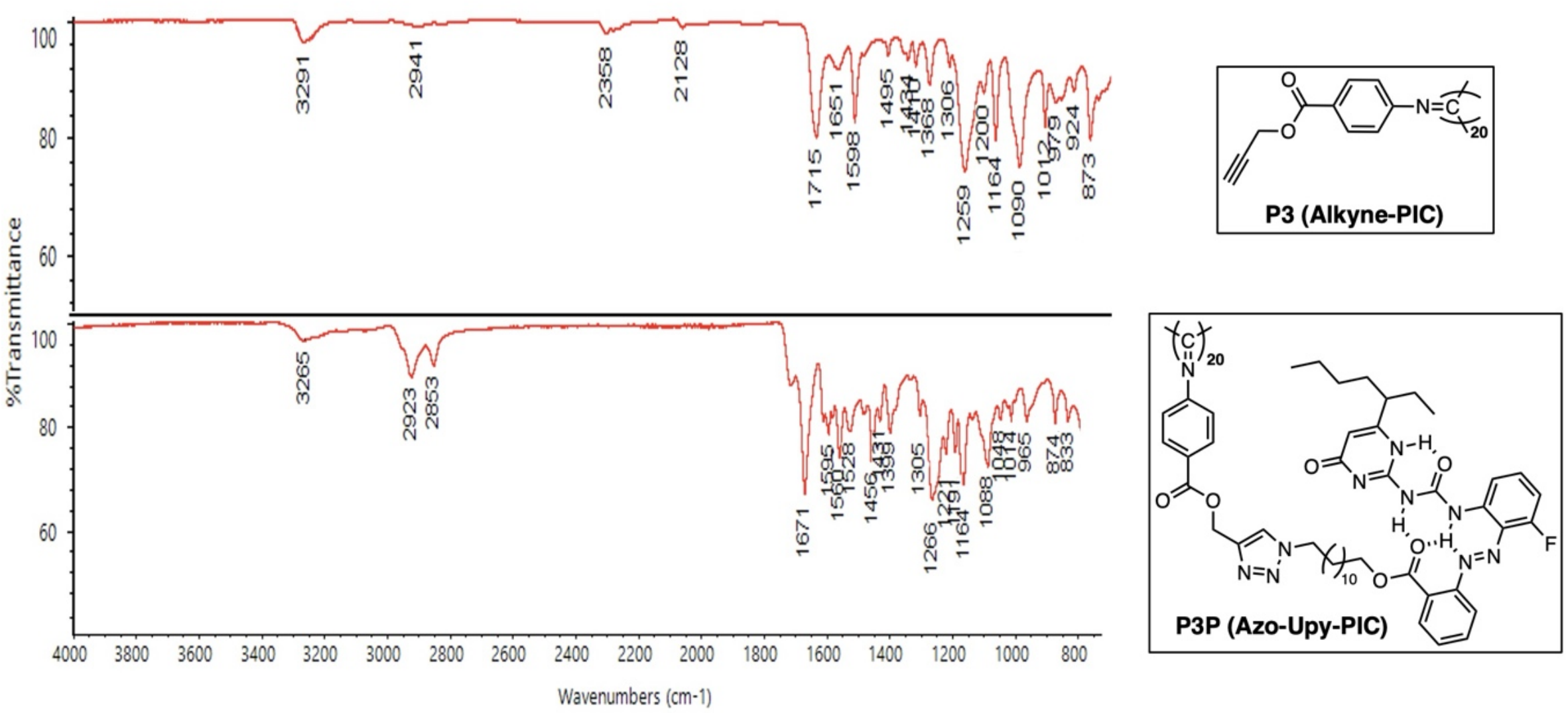

Figure S9. IR spectra of **P3** and **P3P**.

References

(1) Wei, L.; Han, S. T.; Jin, T. T.; Zhan, T. G.; Liu, L. J.; Cui, J.; Zhang, K. D. Towards photoswitchable quadruple hydrogen bonds via a reversible "photolocking" strategy for photocontrolled self-assembly. *Chem. Sci.* **2021**, *12*, 1762-1771.

(2) Xu, A.; Hu, G.; Hu, Y.; Zhang, X.; Liu, K.; Kuang, G.; Zhang, A. Remarkable Structure Effects on Chiroptical Properties of Polyisocyanides Carrying Proline Pendants. *Chem. Asian J.* **2013**, *8*, 2003-2014.

(3) Xue, Y.-X.; Chen, J.-L.; Jiang, Z.-Q.; Yu, Z.; Liu, N.; Yin, J.; Zhu, Y.-Y.; Wu, Z.-Q. Living polymerization of arylisocyanide initiated by the phenylethynyl palladium(II) complex. *Polym. Chem.* **2014**, *5*, 6435-6438.

^{1}H-, ^{13}C-, and ^{19}F- NMR Spectra:

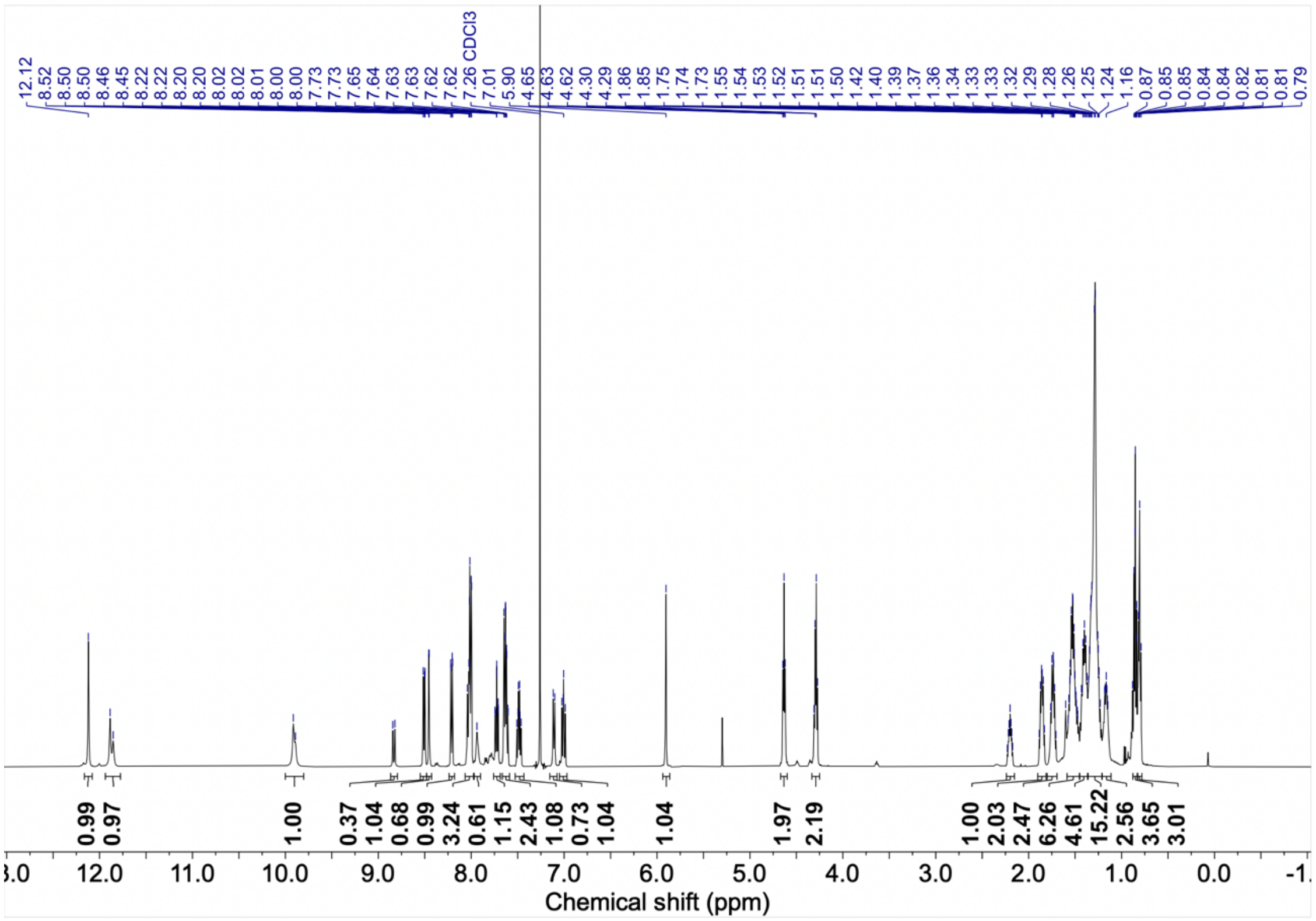

Figure S10. ^{1}H-NMR spectrum of compound **3** (500 MHz, $CDCl_3$, 298 K).

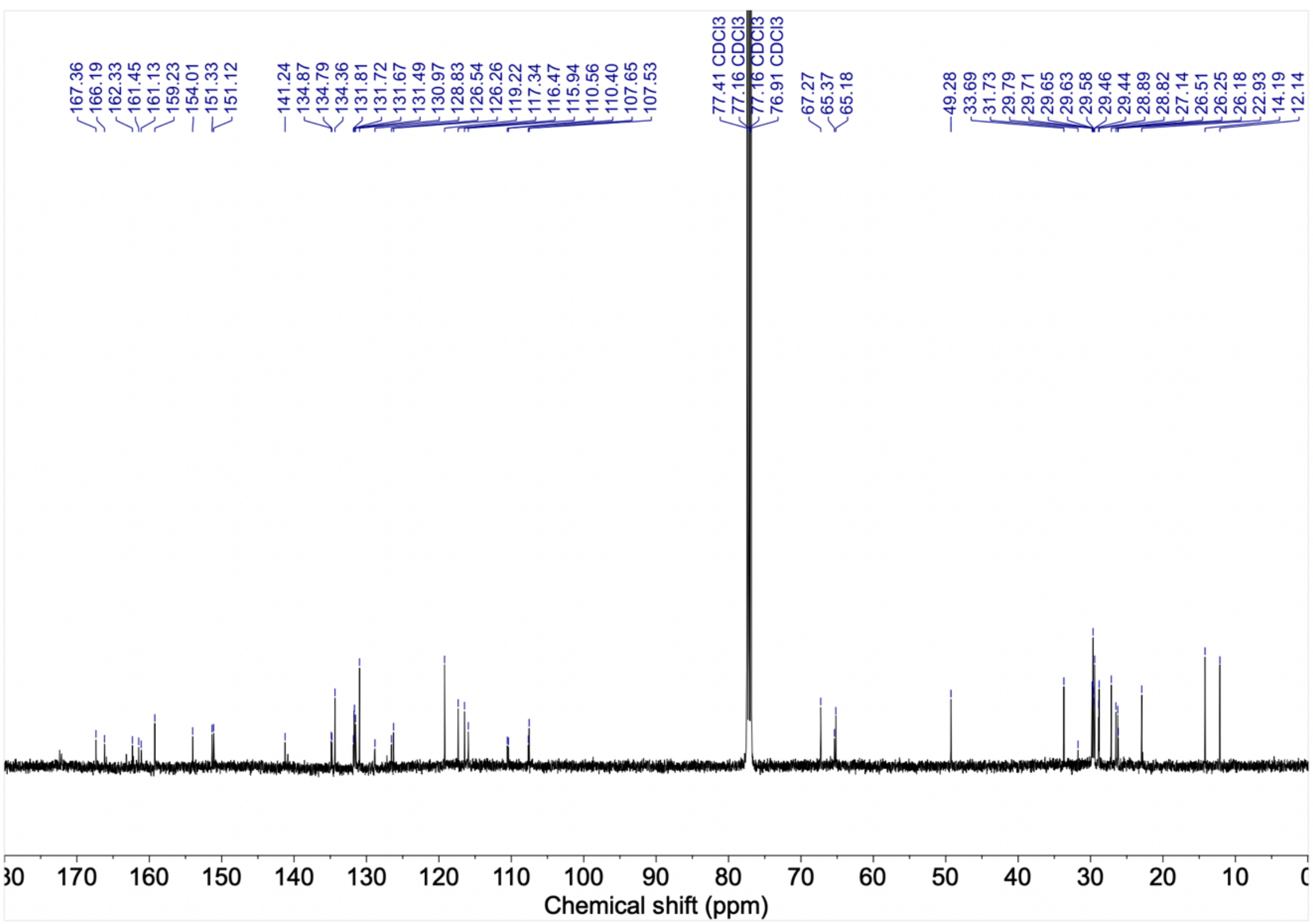

Figure S11. ^{13}C-NMR spectrum of compound **3** (126 MHz, $CDCl_3$, 298 K).

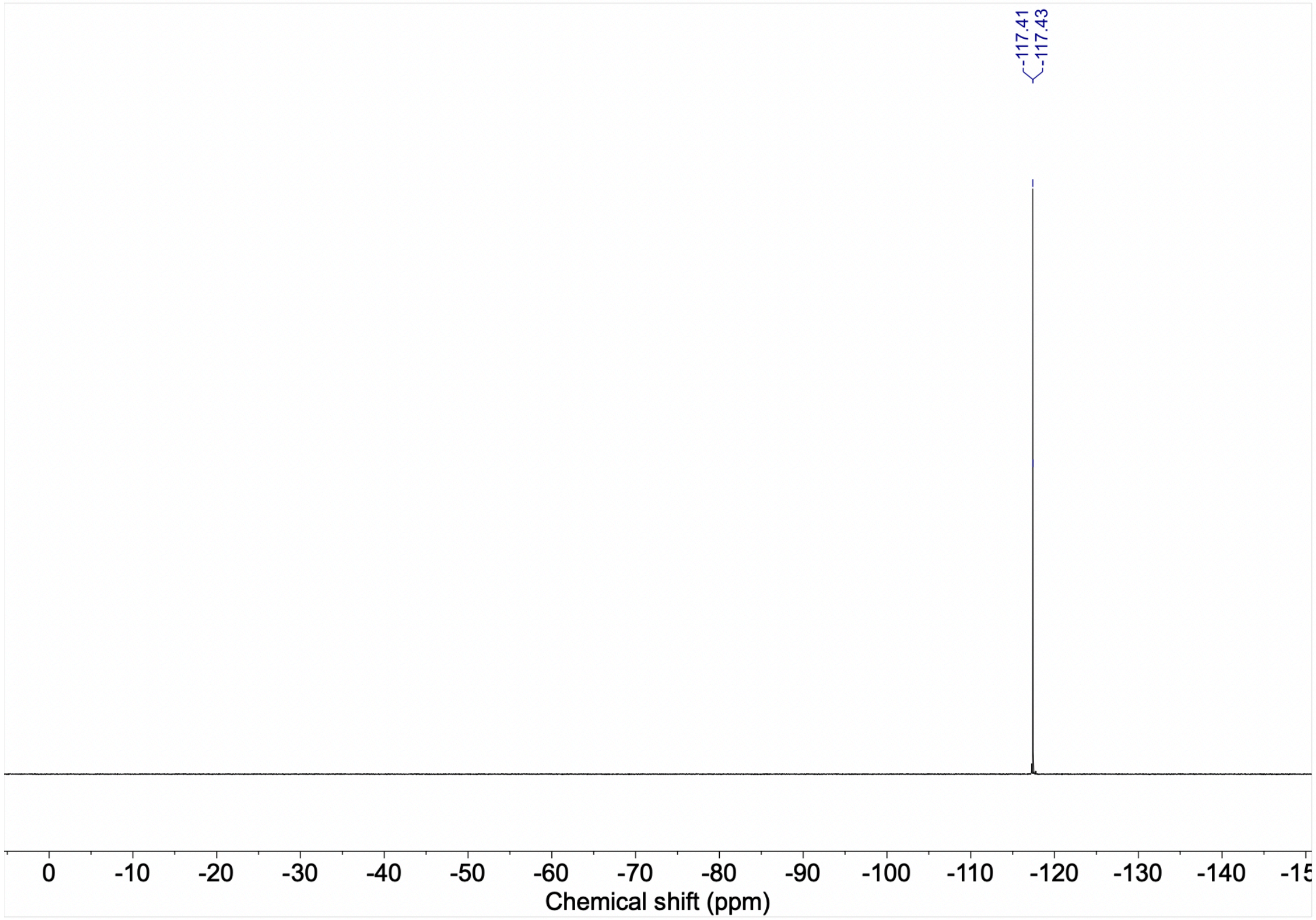

Figure S12. ^{19}F-NMR spectrum of compound **3** (471 MHz, $CDCl_3$, 298 K).

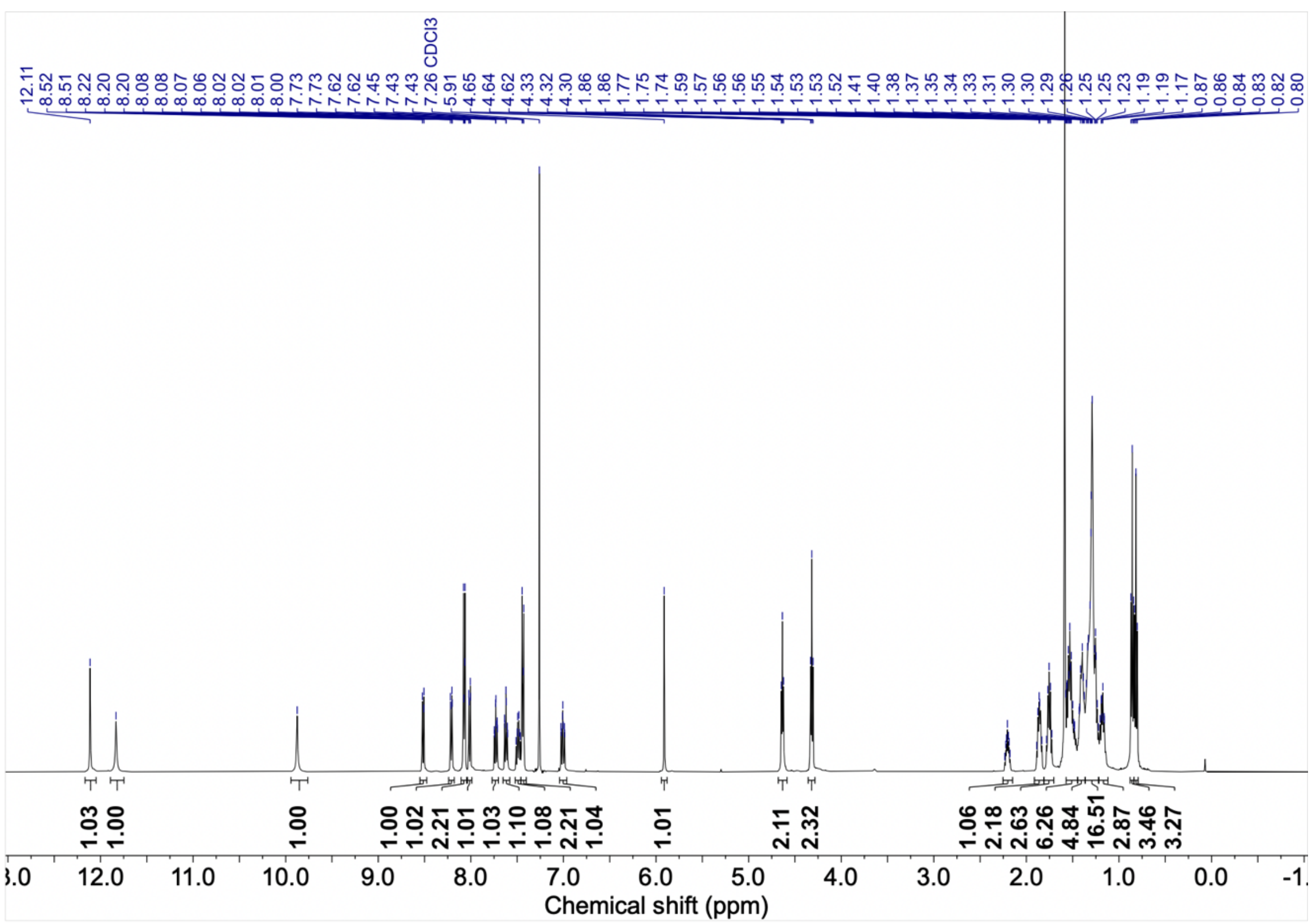

Figure S13. ^{1}H-NMR spectrum of **M1** (**Azo-Upy-PhNC**) (500 MHz, $CDCl_3$, 298 K).

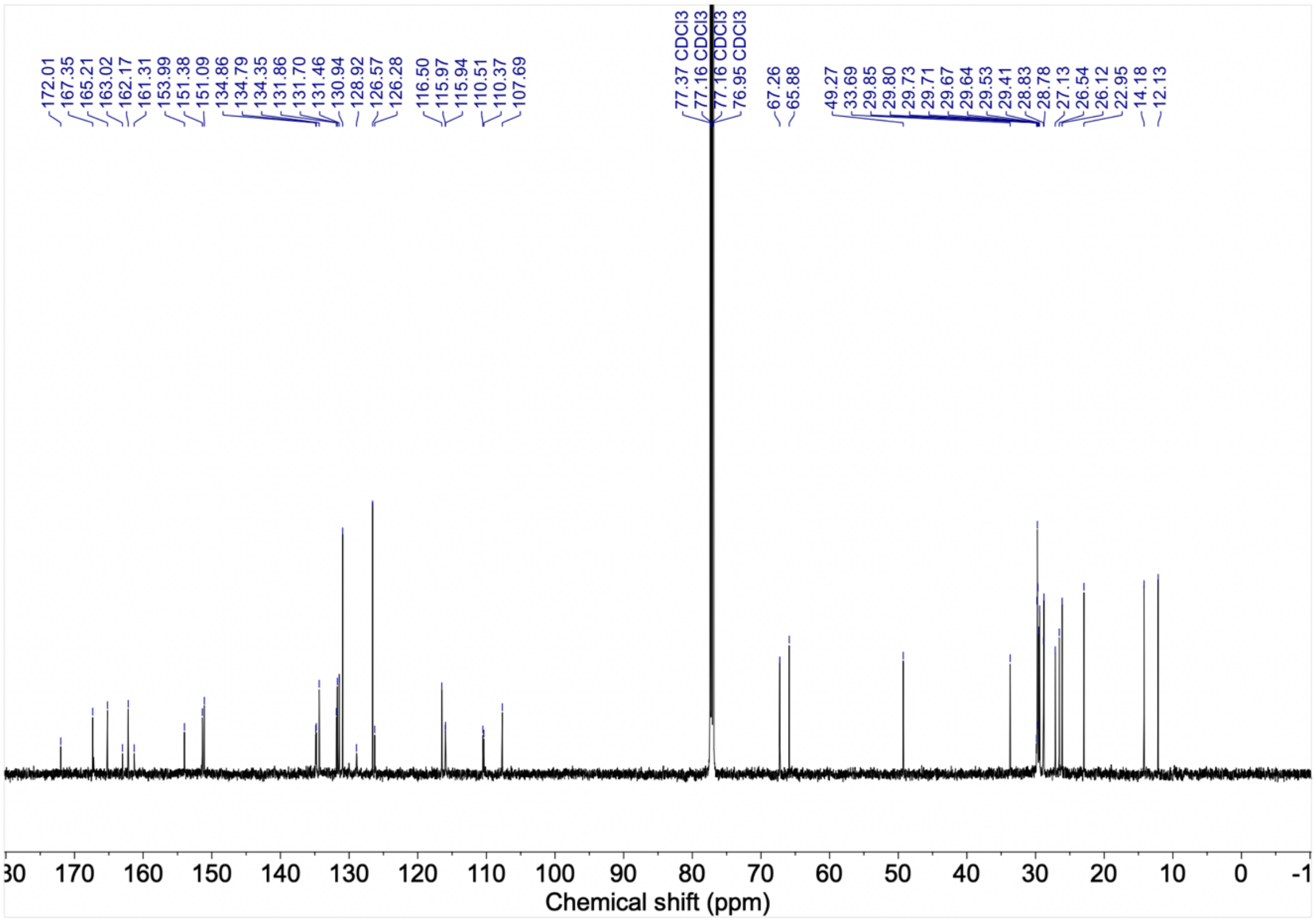

Figure S14. ^{13}C-NMR spectrum of **M1** (**Azo-Upy-PhNC**) (126 MHz, $CDCl_3$, 298 K).

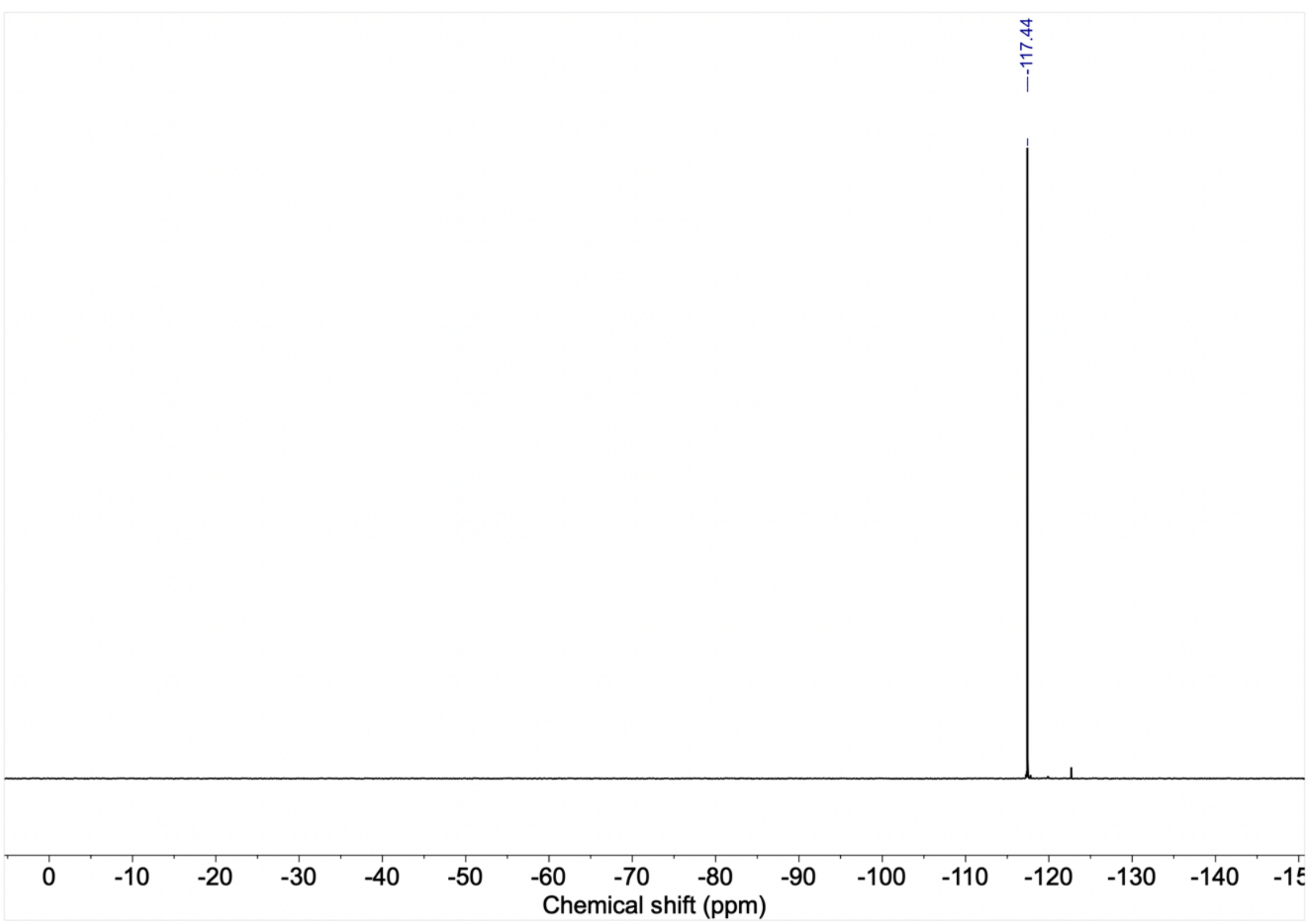

Figure S15. ^{19}F-NMR spectrum of **M1** (**Azo-Upy-PhNC**) (471 MHz, $CDCl_3$, 298 K).

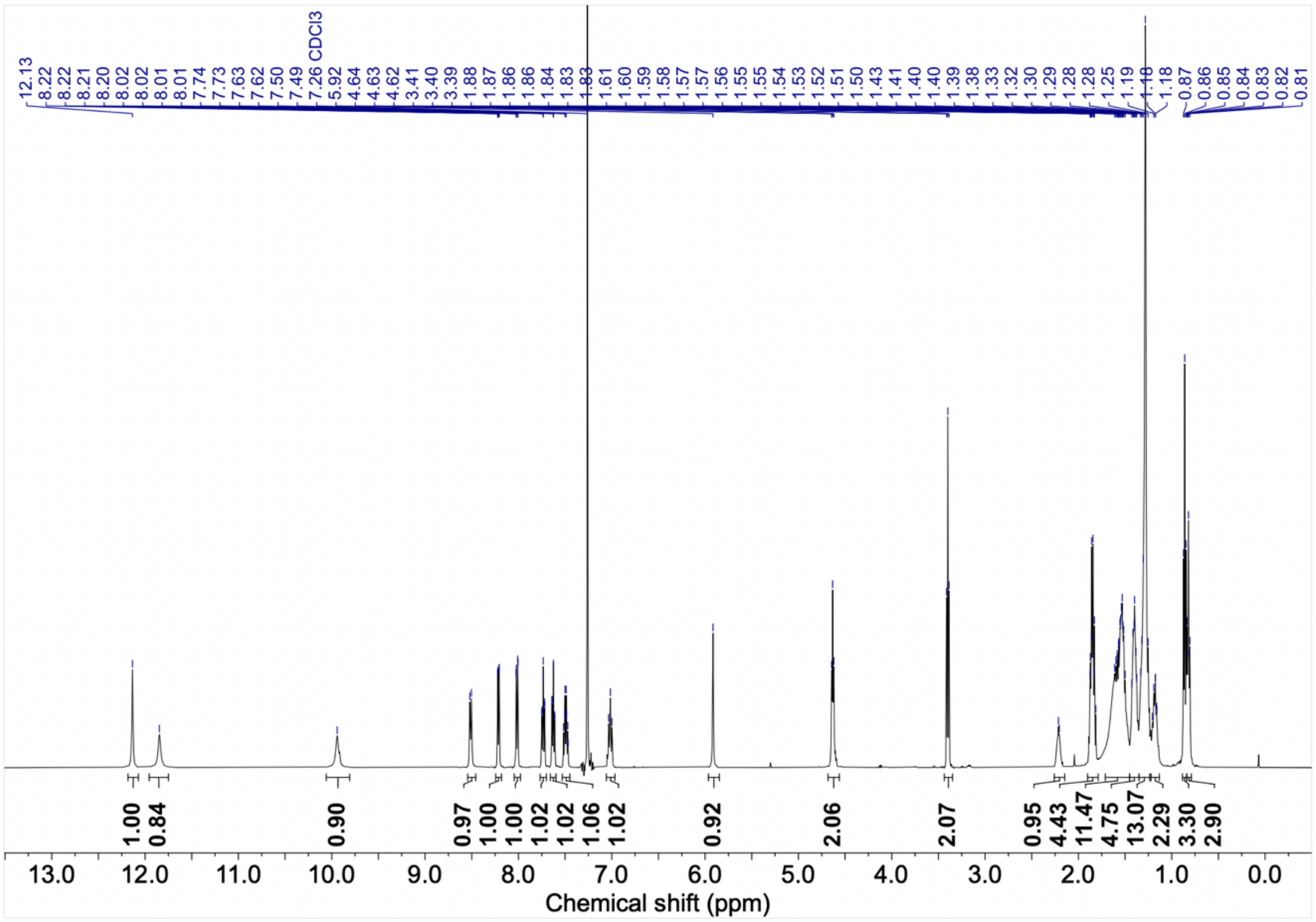

Figure S16. ^{1}H-NMR spectrum of compound **4** (500 MHz, $CDCl_3$, 298 K).

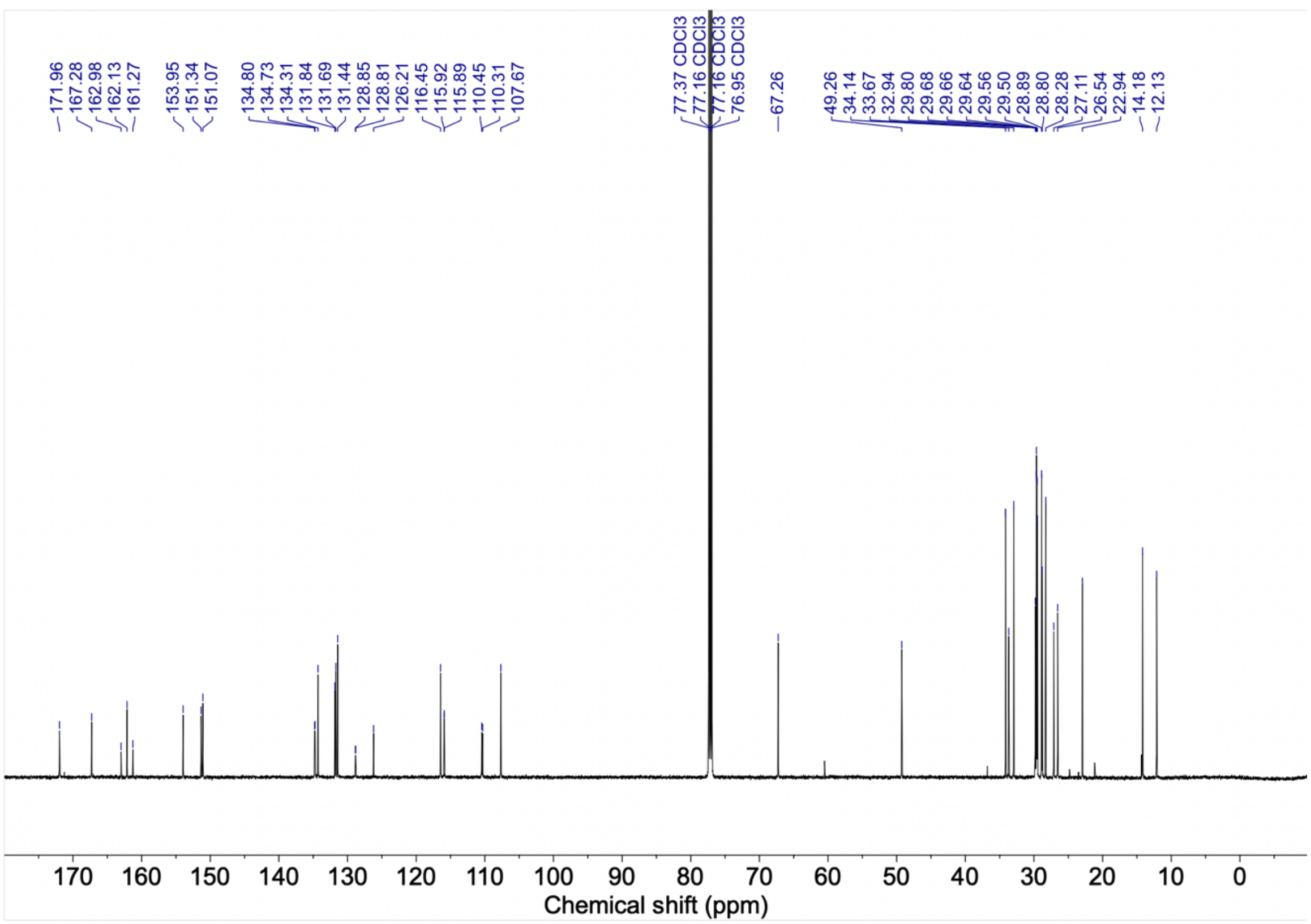

Figure S17. ^{13}C-NMR spectrum of compound **4** (151 MHz, $CDCl_3$, 298 K).

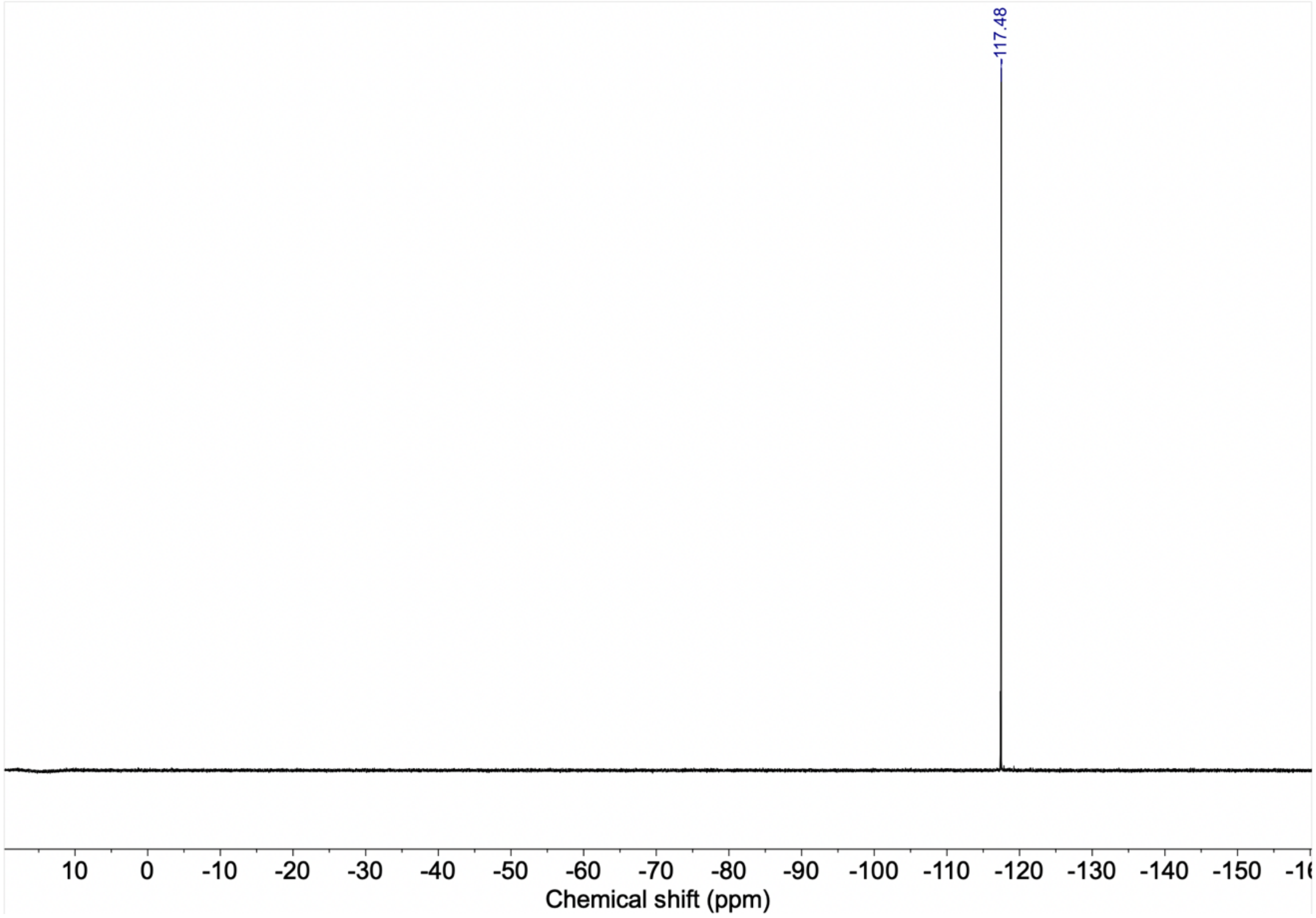

Figure S18. ^{19}F-NMR spectrum of compound **4** (471 MHz, $CDCl_3$, 298 K).

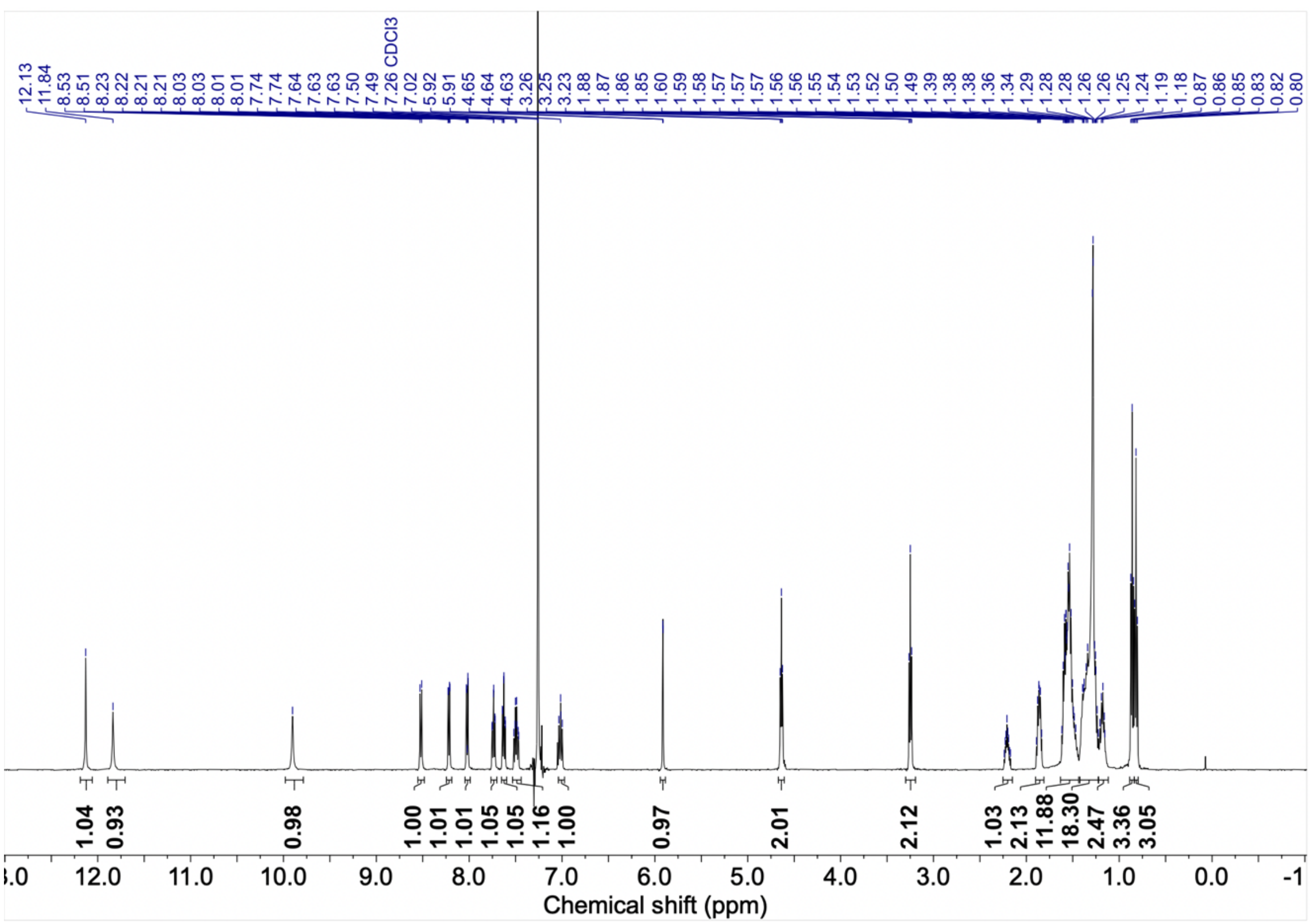

Figure S19. ^{1}H-NMR spectrum of compound **5** (**Azo-Upy-N$_3$**) (500 MHz, $CDCl_3$, 298 K).

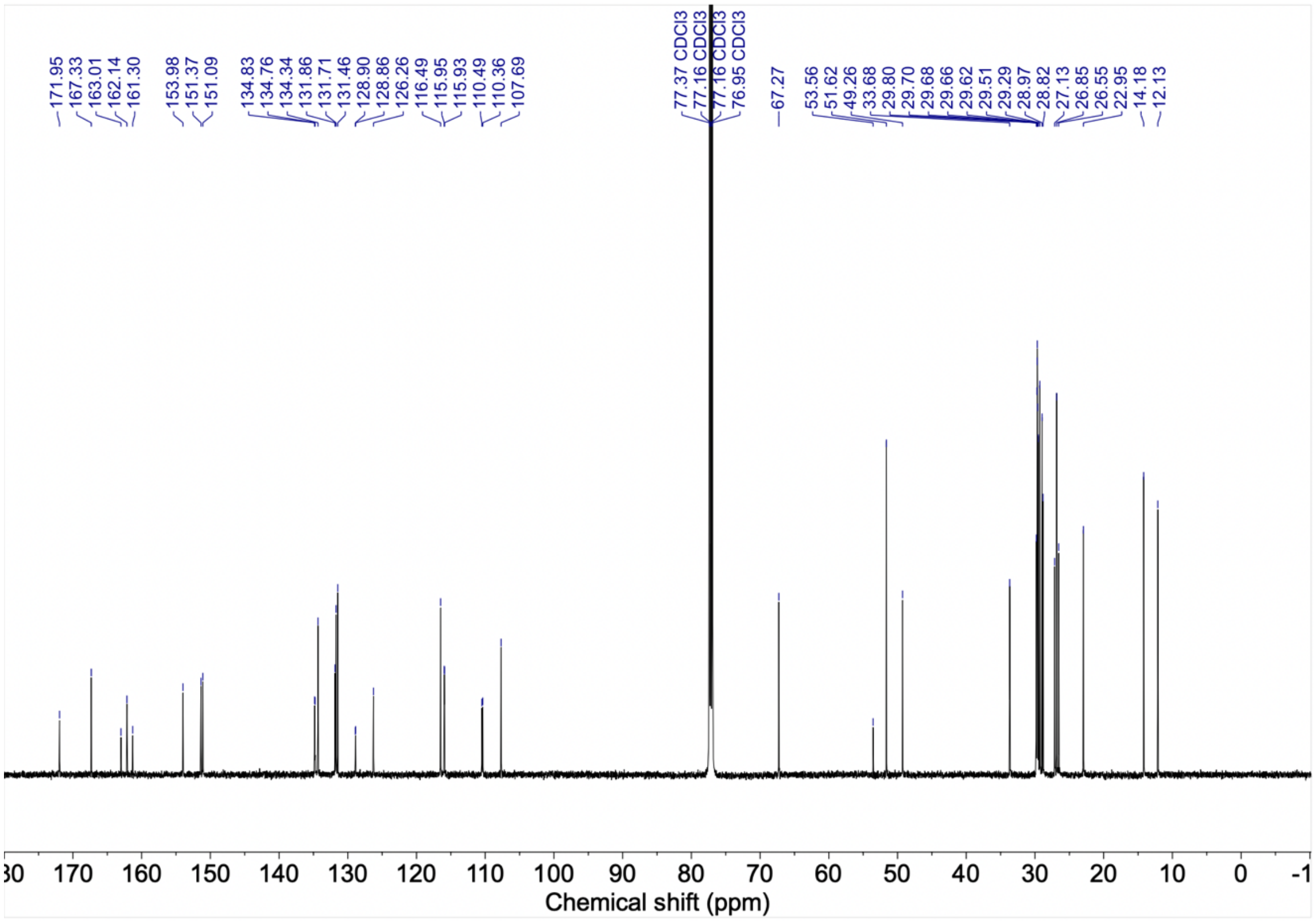

Figure S20. ^{13}C-NMR spectrum of compound **5** (**Azo-Upy-N$_3$**) (151 MHz, $CDCl_3$, 298 K).

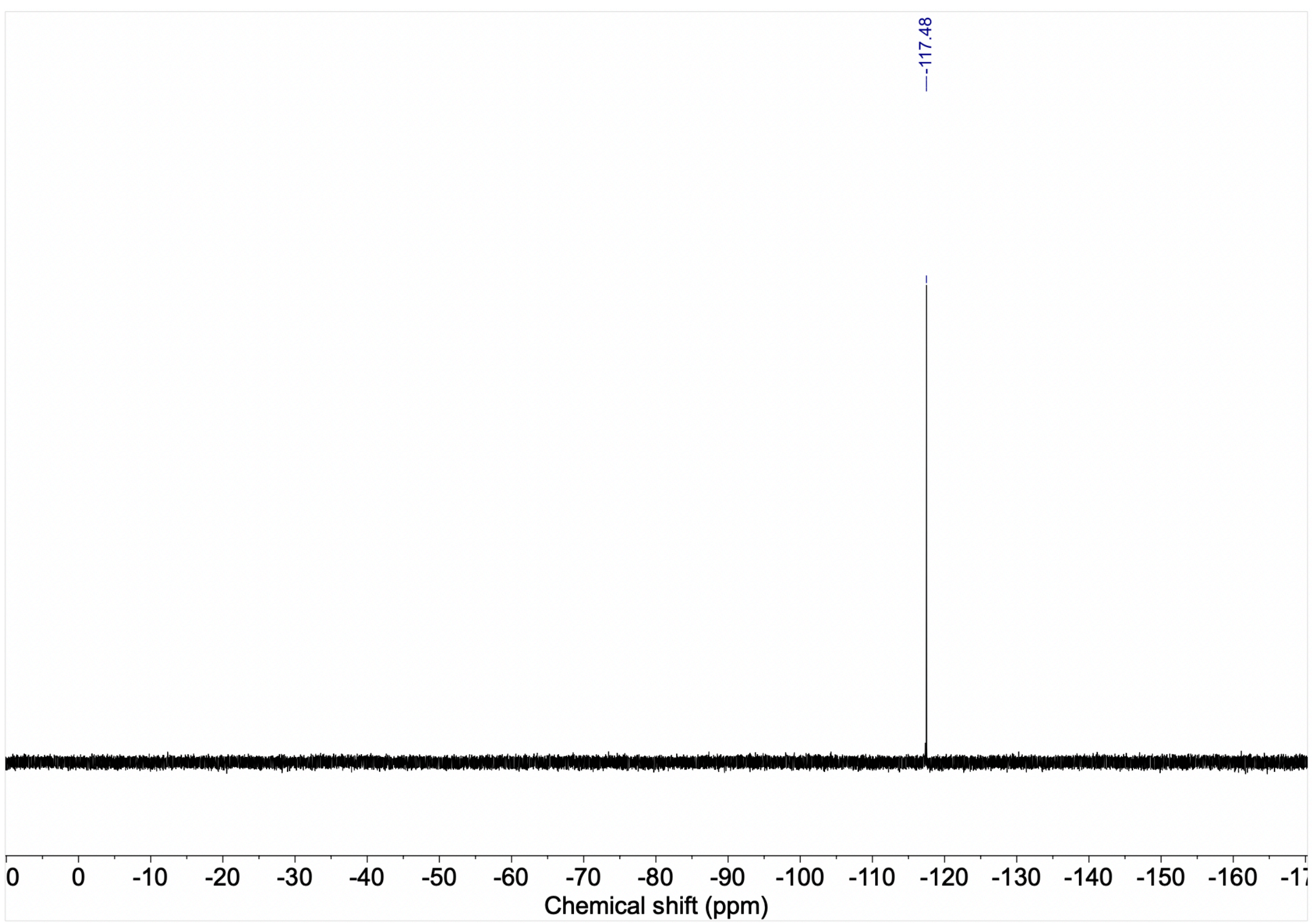

Figure S21. ^{19}F-NMR spectrum of compound **5** (**Azo-Upy-N$_3$**) (471 MHz, $CDCl_3$, 298 K).

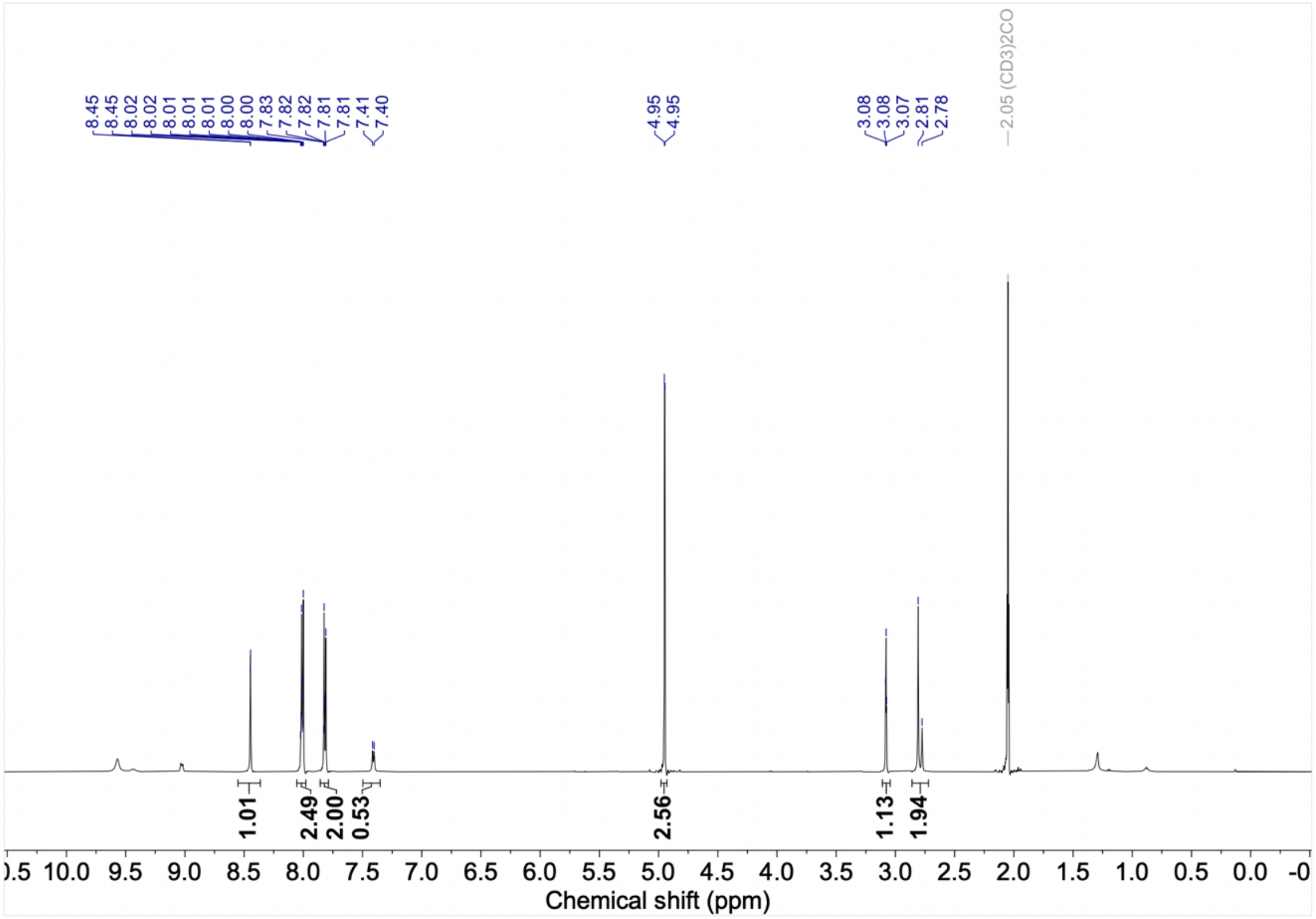

Figure S22. ^{1}H-NMR spectrum of compound **6** (600 MHz, Acetone-d_6, 298 K).

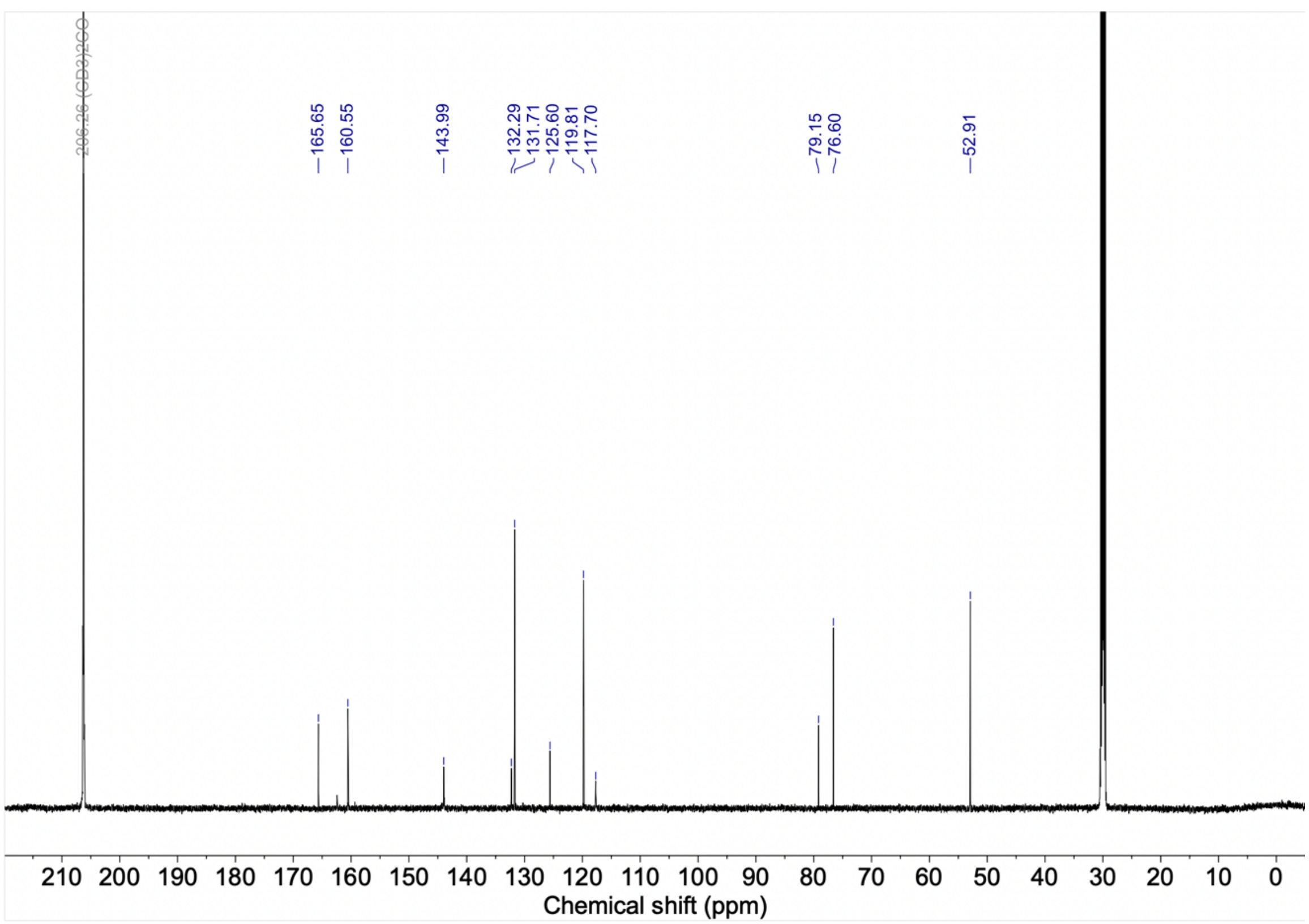

Figure S23. ^{13}C-NMR spectrum of compound **6** (151 MHz, Acetone-d_6, 298 K).

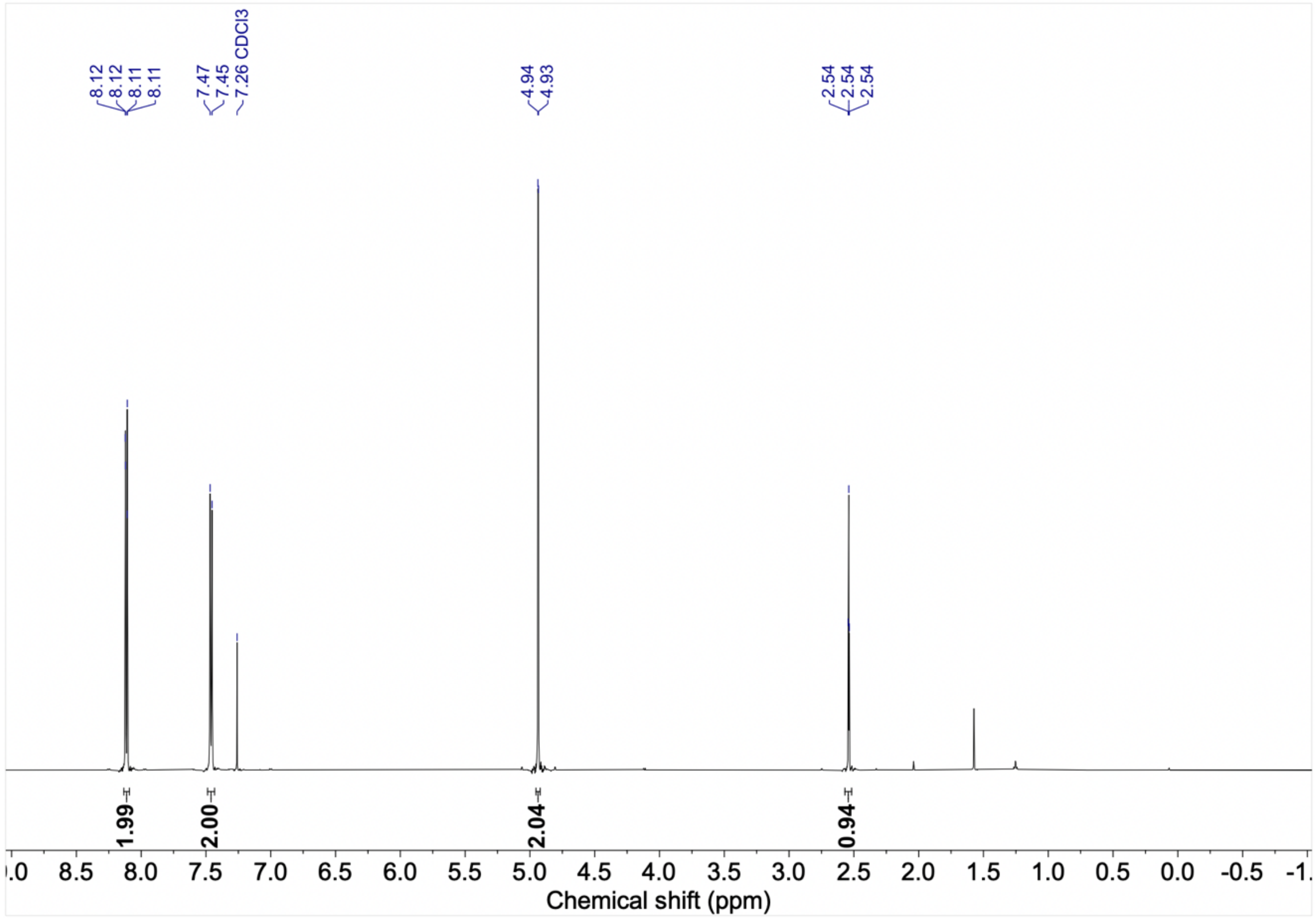

Figure S24. ^{1}H-NMR spectrum of **M3** (**Alkyne-PhNC**) (600 MHz, $CDCl_3$, 298 K).

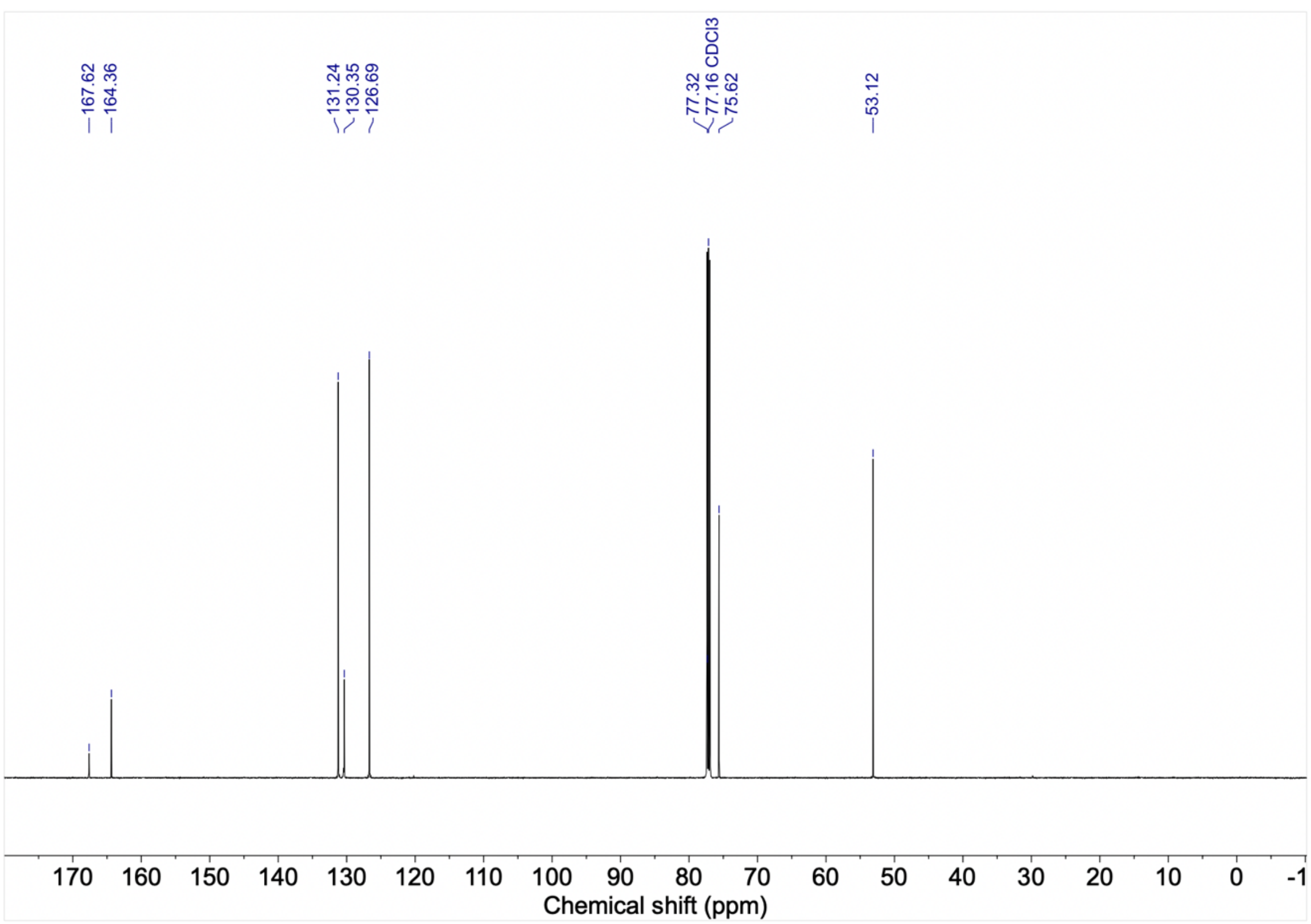

Figure S25. ^{13}C-NMR spectrum of **M3** (**Alkyne-PhNC**) (151 MHz, $CDCl_3$, 298 K).
